# Machine learning reveals biocontrol agents shaping disease outcome in natural *Arabidopsis* populations

**DOI:** 10.1038/s41467-026-75789-w

**Published:** 2026-07-28

**Authors:** Maryam Mahmoudi, Yiheng Hu, Juliana Almario, Paolo Stincone, Lynn-Marie Tenzer, Vasvi Chaudhry, Lukas Braun, Samuel Quinzer, Kay Nieselt, Eric Kemen

**Affiliations:** 1 Microbial Interactions in Plant Ecosystems, IMIT/ZMBP, Eberhard Karls University of Tübingen, Auf der Morgenstelle 32, Tübingen, Germany; 2 Université Claude Bernard Lyon 1, Laboratoire d’Ecologie Microbienne, UMR CNRS 5557, UMR INRAE 1418, VetAgro Sup, Villeurbanne, France; 3 University of Tübingen, CMFI Cluster of Excellence, Interfaculty Institute of Microbiology and Infection Medicine, Tübingen, Germany; 4 Institute for Bioinformatics and Medical Informatics, Eberhard Karls University of Tübingen, Tübingen, Germany; 5 Cluster of Excellence GreenRobust, Eberhard Karls University, Tübingen, Germany

**Keywords:** Microbiome, Plant ecology, Applied microbiology, Machine learning

## Abstract

Plants recruit antagonistic microbes to defend against phytopathogens, offering a route to rational biocontrol beyond empirical screening. Here, using six generations of leaf-microbiome data from natural *Arabidopsis* populations infected by the oomycete *Albugo laibachii*, we show that microbial diversity is driven by infection, site, and host genotype, and that infected plants form modular networks with increased inter-kingdom antagonism. We train four machine-learning models to discriminate infected from uninfected plants by microbiota composition and identify microbes enriched in diseased (disease-associated) or healthy (health-associated) plants. Testing the most predictive bacteria, fungi, and cercozoa *in planta*, we find all confer varying protection against *Albugo*, with health-associated microbes outperforming disease-associated taxa. The best candidate, a *Cystofilobasidium* fungus, is validated in a synthetic community, where genomic and community assays indicate biocontrol acts mainly through microbe-microbe interactions rather than plant immune activation. This work shows that pairing microbiome data with machine learning identifies effective biocontrol agents.

## Abstract (web summary)

Plants can recruit beneficial microbes to suppress pathogens, offering a route to rational biocontrol beyond empirical screening. Here, the authors use machine learning to identify disease-associated and health-associated microbial signatures in Arabidopsis, and test selected microbes for biocontrol activity.

## Introduction

Phytopathogens pose a serious threat to natural ecosystems and crop productivity worldwide^1,2^. For the past five decades, enormous research effort has been devoted to the use of beneficial microbes as biocontrol agents to protect plants from pathogens across diverse crops and natural ecosystems^3^. These microbes operate through multiple mechanisms of action, including competition for nutrients and niches, production of antimicrobial compounds, and induction of host defense responses, highlighting the potential of microbiome-based strategies for sustainable agriculture^4,5^.

In natural ecosystems, plant microbiomes are shaped by a combination of biotic and abiotic factors, including host genotype, as well as temporal and spatial variation^6–9^. However, under pathogen pressure, these relationships can change, and the extent to which such factors influence the microbiome during infection remains unclear. Additionally, in nature, individual plants are often co-infected by multiple pathogens^10^, adding a layer of complexity to plant–pathogen–microbiota interactions. In response to pathogen attack, plants can actively recruit beneficial microbes that enhance resistance or pre-occupy niches to prevent pathogen colonization—a phenomenon known as the “cry for help”^11^. Among these beneficial microbes, some are experimentally supported as biocontrol agents that control disease and promote growth, such as *Trichoderma*^12^, offering eco-friendly alternatives to chemical pesticides^13^. However, developing effective biocontrol agents remains challenging due to inconsistent field performance, limited competitiveness within natural microbiomes, and strong environmental dependency^14,15^. Moreover, current approaches still rely largely on labor-intensive culture-based screening, underscoring the need for more standardized and predictive methods to guide their selection and application in agriculture^16^.

Machine learning has emerged as a powerful tool for analyzing large-scale microbiome data, predicting microbial functions, and identifying key microbes with biocontrol potential for plant health^17,18^. By modeling complex, non-linear relationships within high-dimensional datasets, machine learning algorithms can identify specific microbial signatures that traditional statistical approaches might overlook^19^. For example, machine-learning classifiers have been used to predict soil microbial patterns associated with *Fusarium* wilt disease^20^, link microbiome composition to crop productivity and nitrogen utilization^21^, and identify bacterial strains that suppress *Pseudomonas syringae* infection^22^. Similarly, seed-tuber microbiomes in potato have been used to predict next-season vigor^23^. Machine learning has also been applied to predict soil suppressiveness against fungal pathogen *Rhizoctonia solani* by integrating microbial profiles into interpretable biocontrol indices^24^. While these studies highlight the potential of machine learning for predicting plant health from microbiome data, relatively few have used these insights to identify and experimentally validate novel biocontrol candidates from multi-kingdom natural microbiomes.

In this study, we investigate how natural microbiome dynamics contribute to plant protection by identifying microbes with biocontrol potential. We analyze six years of leaf microbiome data from a natural *Arabidopsis thaliana* metapopulation in southern Germany, where the oomycete *Albugo laibachii* persists as a core member of the leaf community^9^. We also examine how microbiome composition and structure were shaped by host genotype, site, and multi-kingdom microbial interactions under *Albugo* infection. Using machine learning, we identify microbial taxa associated with *Albugo*-infected and uninfected plants. We then isolate and experimentally validate candidate microbes for their biocontrol activity against *Albugo laibachii*, both individually and within a synthetic community. Further genomic and host defense response analyses are performed on the most important selected candidate to uncover mechanisms underlying plant protection. This approach provides a framework for integrating natural microbiome data with machine learning to identify and test biocontrol agents that enhance plant resilience to pathogens.

## Results

### *Albugo* infection reduces the diversity of microbial community in the phyllosphere of *A. thaliana*

**Fig. 1:**
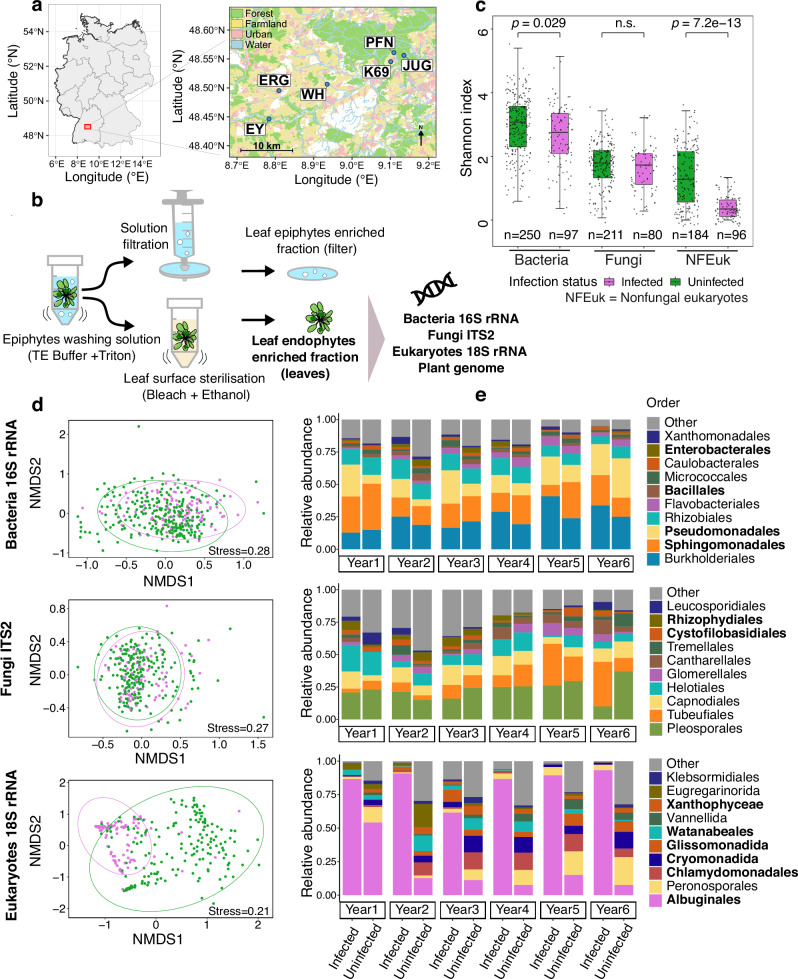
Diversity and composition of leaf microbial communities in *Albugo*-infected and uninfected plants. **a** Map showing the six sampling locations of natural *A. thaliana* in southern Germany near Tübingen. **b** Leaf epiphytic and endophytic fractions collected from each sampled rosette. Microbiome analysis used Illumina amplicon sequencing (MiSeq, 2 × 300 bp) targeting bacterial 16S rRNA V5–V7, fungal ITS2, and eukaryotic 18S rRNA V9 regions. Whole-genome sequencing of the endophytic fraction was performed on an Illumina NovaSeq platform (2 × 150 bp). (image is adapted from^9^). **c** Microbial alpha-diversity is measured by Shannon’s H index in bacterial, fungal, and nonfungal eukaryotic (NFEuk) communities. Box plots display individual samples as dots. Exact *p* values from two-sided Tukey’s HSD tests comparing infected and uninfected samples within each community are shown (n.s., not significant). **d** Separation of infected (purple) and uninfected (green) samples using non-metric multidimensional scaling analysis (NMDS) based on Bray-Curtis dissimilarities. Each dot represents a single sample. **e** Average relative abundance of bacterial, fungal, and NFEuk orders, grouped by plant infection status and sampling years. Taxa exhibiting significant differences between infected/uninfected groups are indicated in bold (Wilcoxon test, *p* < 0.05; see also Supplementary Fig. 1). In all box plots, the center line denotes the median, box bounds the 25th and 75th percentiles, and whiskers the most extreme values within 1.5× the interquartile range; overlaid dots are individual samples, and the n values below each box indicate the number of biologically independent leaf samples. Source data are provided as a Source Data file.

To investigate the diversity and compositional dynamics of the phyllosphere microbiome in relation to leaf infection by the obligate biotrophic pathogen *Albugo* (Oomycota phylum), we obtained the microbiome data from an *A. thaliana* collection, on which we have previously performed amplicon sequencing and described the microbiome^9^. Shoot samples (*n* = 351) were collected from six sites near Tübingen, Germany (48.52° N, 9.06° E, Fig. 1a), over six years (2014–2019). Microbiome data were obtained from epiphytic and endophytic leaf fractions using amplicon sequencing, and the host genotype was determined using whole genome sequencing (Fig. 1b)^9^. Here, we further analyzed endophytic microbiome due to their tight association with the host^25^. Since *Albugo* was the major pathogen in *A. thaliana* leaves at the time of sampling, samples were categorized as infected with observable symptoms or uninfected without observable symptoms based on the presence of characteristic white blisters on leaves (Supplementary Table 1).

We first conducted diversity analysis to compare the leaf-associated microbial communities between infected and uninfected plants. Alpha diversity (within-sample diversity, measured by Shannon’s index) demonstrated that, on average, bacterial and nonfungal eukaryotic (NFEuk) communities in infected plants were 1.1-fold and 2.8-fold less diverse, respectively, than those in uninfected plants (Tukey’s HSD test, *p* < 0.05). However, no significant differences were observed in fungal communities (Fig. 1c). A stratified permutational multivariate analysis of variance (PERMANOVA) conducted within each sampling year revealed that infection status explained on average 4.7% of the variation in bacteria, 4.3% in fungi, and 15.1% in NFEuk (Supplementary Data 1). These variations were further visualized using non-metric multidimensional scaling (NMDS), which revealed that the infected plants were most clearly separated from the uninfected groups in the NFEuk communities, followed by bacteria and less so in the fungal communities (Fig. 1d).

Differences between leaf-microbial communities of infected and uninfected plants were associated with the enrichment of major microbial orders over the sampling years (Fig. 1e, Supplementary Fig. 1). Among bacteria, microbial orders such as *Sphingomonadales* were enriched 1.3-fold in uninfected plants. This order is known for containing members that have been demonstrated to be beneficial for plant health and productivity^26^. Whereas, *Pseudomonadales* enriched 1.5 times in infected samples (comparison of mean relative abundances; Wilcoxon test, *p* < 0.05). Among fungi, Basidiomycete yeast *Cystofilobasidiales* exhibited a slightly higher abundance in uninfected plants (1.07 times higher), and *Rhizophydiales* were enriched 1.9 times in infected plants. In NFEuk, *Albuginales* (*Albugo* lineage) were significantly enriched and dominated the community in infected plants (6.7-fold increase), corresponding to their role in distinguishing infection status among samples. In contrast, the relative abundance of several other NFEuk orders was largely enriched in uninfected plants, including green algae lineages *Watanabeales*, *Xanthophyceae* and *Chlamydomonadales* (5.9–14.5 times), and *Cercozoa* lineages *Cryomonadida* and *Glissomonadida* (7.6–26.4 times). Together with the observations from diversity analysis these results indicate that infection status significantly reduced the species richness of the *A. thaliana* endophytic microbial community and altered the relative abundance of key microbial lineages.

### Contributions of host genotype and sampling site to microbiome variation under *Albugo* infection

**Fig. 2:**
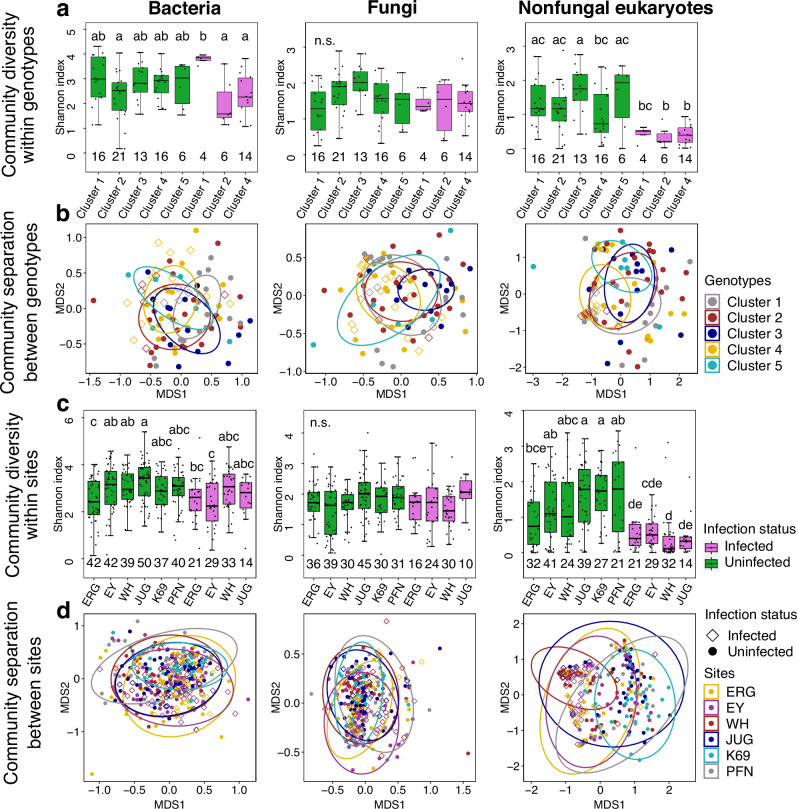
Diversity and separation of leaf microbial communities in infected and uninfected plants across different genotypes and sampling sites. Alpha diversity, measured by Shannon’s H index, represents the within-sample diversity of microbial communities across infected and uninfected samples from different genotype clusters (**a**) and sampling sites (**c**). Statistically significant differences between groups are indicated by different letters (2 sided Dunn test, *p* < 0.05). **b** NMDS plots, based on Bray-Curtis dissimilarities, show the separation between infected and uninfected samples across genotype clusters, while (**d**) displays the separation by sampling sites. In all box plots, the center line denotes the median, box bounds the 25th and 75th percentiles, and whiskers the most extreme values within 1.5× the interquartile range; overlaid dots are individual samples, and the n values below each box indicate the number of biologically independent leaf samples. Source data are provided as a Source Data file.

If infection contributes significantly to microbial variation, we would expect the relative contributions of host genotype and sampling site to microbial community composition to differ between infected and uninfected plants. To examine this, we analyzed the relative contributions of host genotype and sampling site to the diversity and composition of the leaf microbiome in both infected and uninfected plants. Host genotypes were categorized into five clusters based on a previous whole-genome single nucleotide polymorphism analysis^9^. Notably, genotype information was available for only 96 of the 351 samples (Supplementary Table 1), indicating that these analyses represent a subset of the total dataset and may not fully capture all site- or infection-associated effects. Three of these clusters (clusters 1, 2, and 4) included *Albugo*-infected plants. Overall, infection status was associated with a modest yet significant reduction in nonfungal eukaryote (NFEuk) alpha diversity (Fig. 2a). However, patterns within specific clusters varied: among infected plants, cluster 1 exhibited comparatively higher bacterial diversity than other infected clusters, suggesting that certain bacterial communities may be less affected by infection. Conversely, within uninfected plants, cluster 3 displayed greater NFEuk diversity than cluster 4, indicating cluster-specific differences in community structure. PERMANOVA revealed that host genotype significantly shaped community composition (beta-diversity), explaining 6.0% and 10.9% of the variation in bacterial and NFEuk communities (Supplementary Data 1). Stratified analyses of infection status within each genotype showed that infection explained 10.1% and 32.0% of the variation in bacterial and NFEuk communities (Supplementary Data 1), indicating that the impact of *Albugo* infection differs among host genotypes. These genotype- and infection-associated differences were further visualized using NMDS ordination (Fig. 2b).

Sampling site also emerged as a major driver of both alpha- and beta-diversity. Two sites (K69 and PFN) contained exclusively uninfected plants, while four (EY, WH, JUG, and ERG) contained a mix of infected and uninfected individuals (Fig. 2c). Within these mixed sites, infected plants generally had lower bacterial and NFEuk alpha-diversity than their uninfected counterparts (Dunn test, *p* < 0.05). We also observed site-specific variation. For example, uninfected plants at site ERG had lower alpha-diversity than JUG, EY and WH for bacteria, and lower than JUG and K69 in NFEuk (Fig. 2c). Overall, sampling site explained 6.0–11.7% of the variation in microbial community composition (Supplementary Data 1, Fig. 2d). When stratifying by infection status within sites, the explained variation was on average 3.6% for bacteria, 2.6% for fungi, and 13.4% for NFEuk, indicating that spatial heterogeneity among sites contributes to infection-associated community shifts. The effects of host genotype were largely consistent across sites, and PERMANOVA did not detect significant site-by-genotype interactions (Supplementary Data 1), suggesting that genotype-driven microbiome differences act at a broader metacommunity scale rather than through local, site-specific adaptation.

In summary, these findings demonstrate that both host genetic background and site-dependent environmental factors influence the leaf microbiome and jointly modulate the impact of *Albugo* infection on community composition.

### *Albugo* infection modulates cross-kingdom leaf microbial networks

**Fig. 3:**
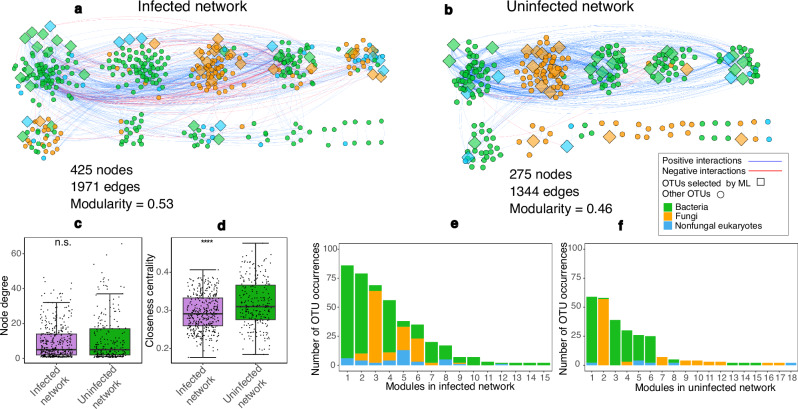
Changes in microbial co-abundance networks of infected and uninfected plants. Co-abundance networks are shown for infected (**a**) and uninfected (**b**) plants. Nodes represent OTUs, and edges (connections between OTUs) indicate correlations ( | *r* | ≥ 0.2 and *p* ≤ 0.01). Nodes are color-coded by microbial taxa and clustered into modules. Microbes identified by machine learning analysis (Fig. 5) are indicated with diamond shapes. **c**, **d** Node degree distribution and node closeness centrality. Significance values indicate group differences based on the Wilcoxon test (****p ≤ 0.0001). Each point represents an OTU in the network. **e**, **f** Histograms illustrate the distribution of OTUs among modules. OTU taxonomy at the kingdom level is indicated. In the box plots (**c**, **d**), the center line denotes the median, box bounds the 25th and 75th percentiles, and whiskers the most extreme values within 1.5× the interquartile range; each dot represents one OTU (network node), with *n* = 425 OTUs in the infected and 275 in the uninfected network. Source data are provided as a Source Data file.

To dissect the effect of *Albugo* infection on the cross-kingdom interactions and ecological connectivity of the leaf microbiome, we constructed cross-kingdom co-abundance networks from the leaf microbiomes of infected and uninfected plants and compared their structural properties. We selected correlation thresholds for network construction by examining network properties and applied an absolute cutoff of |*r* | ≥ 0.2 and *p* ≤ 0.01 to preserve robust structure (Supplementary Fig. 2a–e). The microbial network inferred from infected plants contained substantially larger network size, with 1.55 times as many nodes (425 vs. 275) and 1.47 times as many edges (1971 vs. 1344) compared to the uninfected network (Fig. 3a, b). While the average OTU degree, representing the number of connections per OTU was similar between networks, the infected network showed lower average closeness centrality, indicating greater average path lengths between nodes thus lower inter-connectivity (Fig. 3c, d).

To assess whether observed shifts in microbial interactions lead to niche partitioning, we quantified network modularity^27^. Modularity identifies clusters of OTUs with high internal connectivity that may represent functionally related taxa^28–30^. The infected network exhibited higher modularity (0.53 vs. 0.46; Fig. 3a, b), associated with more medium-to-large modules (8 modules vs. 6 modules with >10 OTUs; Fig. 3e, f) compared to the uninfected network. These characteristics indicate that microbial networks from infected plants are more fragmented, with more connections within modules than between modules.

Since network modules were highly enriched in same-kingdom nodes (Fig. 3e, f), we next examined the impact of *Albugo* infection on intra- and inter-kingdom interactions. We compared positive and negative correlations within kingdoms (bacteria–bacteria, fungi–fungi, NFEuk–NFEuk) and between kingdoms (bacteria–fungi, bacteria–NFEuk, fungi–NFEuk) (Supplementary Fig. 3a, b). While both networks contained predominantly positive correlations, the infected network displayed a higher proportion of negative edges in inter-kingdom interactions compared to the uninfected network (bacteria–fungi: 13% vs. 5%; bacteria–NFEuk: 32% vs. 4%; fungi–NFEuk: 18% vs. 0%) (Supplementary Fig. 3c, d), indicating increased cross-kingdom antagonism under *Albugo* infection. Collectively, these results suggest that *Albugo* infection triggers a systemic reorganization of the leaf microbial network, enhancing modularity and promoting antagonistic interactions across kingdoms.

### Accurate microbiome-based prediction of plant infection status using machine learning models

Given that *Albugo* infection resulted in a systematic reorganization of the leaf microbiome, we hypothesized that these different microbial communities could serve as a robust indicator, enabling accurate prediction of plant infection status from microbiome data. To test this hypothesis, we employed four machine learning classifiers—Random Forest (RF), Support Vector Machine (SVM), Logistic Regression (LR), and Multilayer Perceptron (MLP)—to screen and identify microbial features (OTUs) predictive of plant infection status. These models were selected based on their ability to capture both linear (SVM and LR) and non-linear (RF and MLP) relationships within sparse microbiome data^31^. Specifically, RF and SVM are robust to overfitting in high-dimensional settings; LR provides an interpretable linear baseline; and MLP can model complex non-linear feature interactions^32^.

**Fig. 4:**
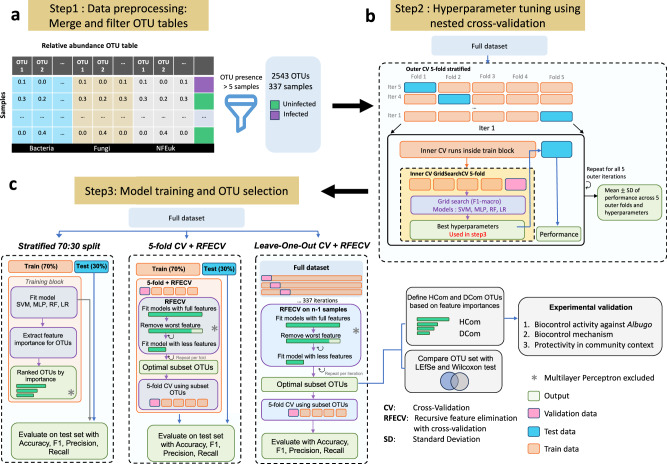
Classification of plant infection status and feature selection using machine learning classifiers. **a** Bacterial, fungal, and NFEuk OTU tables were converted to relative abundance within each kingdom and merged. OTUs present in fewer than five samples were removed, yielding 2543 OTUs across 337 samples. Samples were classified as infected (purple) or uninfected (green) based on *Albugo* presence. **b** Hyperparameters for Support Vector Machine (SVM), Random Forest (RF), Logistic Regression (LR), and Multilayer Perceptron (MLP) classifiers were optimized using nested stratified 5-fold cross-validation with GridSearchCV and macro-averaged F1-score (Supplementary Table 2). Optimized hyperparameters were fixed for all downstream analyses (**c**). **c** Four classifiers were trained and evaluated using three complementary approaches: a stratified 70:30 train–test split (Approach 1), 5-fold cross-validation with RFECV (Approach 2), and leave-one-out cross-validation (LOO CV; Approach 3). Feature importance for key OTU selection was determined from the 70% training set in Approach 1 and by recursive feature elimination with cross-validation (RFECV) in Approaches 2 and 3. OTUs identified through LOO CV were taxonomically confirmed by BLAST and compared with LEfSe and Wilcoxon test results. Selected OTUs were subsequently used for experimental validation. MLP was excluded from RFECV-based approaches. Colored boxes indicate train data (orange), test data (blue), validation data (pink), and output (light green).

Prior to model training, OTU tables from bacteria, fungi, and NFEuk were independently converted to relative abundance within each kingdom and merged into a single composite table. OTUs present in fewer than five samples were removed, resulting in 2,543 OTUs across 337 samples, each labeled as infected or uninfected based on the presence of white rust caused by *Albugo* (Fig. 4a). To maximize predictive performance and ensure an unbiased comparison, we optimized hyperparameters using a nested cross-validation framework, employing an inner GridSearchCV loop for parameter identification and an outer loop for performance assessment (Fig. 4b, Supplementary Table 2).

We then sought to train the selected classifiers with three complementary approaches and assess their performance and identify informative OTUs (Fig. 4c). In the first approach, we first performed a stratified 70:30 train-test split, with 169 uninfected and 66 infected samples in the training set, and 73 uninfected and 29 infected samples in the test set. Feature importance was assessed using the mean decrease in impurity for the RF, absolute coefficients for the SVM (linear kernel), and standardized coefficients for LR. In the second approach, following a stratified 70:30 train–test split, recursive feature elimination with cross-validation (RFECV) was applied to the training set to identify informative OTUs. Model performance was then evaluated using stratified 5-fold cross-validation on the training data for both full and reduced feature sets, followed by assessment on the independent test set. Subsequently, we used a leave-one-out cross-validation (LOO CV) strategy to identify the optimal subset of OTUs, validating the final model performance through stratified 5-fold cross-validation.

Across all evaluation schemes, performance metrics varied among classifiers (Supplementary Fig. 4a). SVM achieved consistently stable performance, with accuracy of ~85% and F1-scores around 0.81–0.82 across all data-splitting strategies. MLP showed similarly or slightly higher performance (accuracy up to ~87%, F1 ~ 0.85), whereas LR performed comparably with slightly higher variability across evaluation schemes. In contrast, RF displayed the greatest variability, with performance ranging from lower accuracy ( ~ 75%) and F1 ( ~ 0.57) in the train–test setting to substantially improved performance under cross-validation schemes (accuracy up to ~91%). Receiver operating characteristic (ROC) analyses further showed that SVM and LR achieved the highest and comparable area under the curve (AUC) values (~0.93) followed closely by MLP ( ~ 0.92), while RF showed comparatively lower performance (AUC ~ 0.86). These results were obtained from the 70:30 train–test split (Supplementary Fig. 4b).

While each classifier exhibited strengths and limitations depending on the data-splitting strategy, feature selection using LOO-based RFECV combined with cross-validation yielded the most robust performance across all classifiers. Among the four classifiers, SVM demonstrated the most consistent and balanced performance in predicting *Albugo* infection status, whereas RF achieved the highest performance specifically under the LOO-based evaluation scheme. Overall, these results demonstrate that machine learning can successfully predict plant infection status, highlighting that microbiome shifts serve as robust indicators of *Albugo* infection.

### Machine learning-based identification of leaf microbial taxa critical for *Albugo* infection

Next, we aimed to identify microbial taxa most informative for predicting *Albugo* infection. We examined feature importance derived from machine learning models across all three evaluation schemes (train-test split, 5-fold cross-validation [5-fold CV], and LOO CV) (Fig. 4c). The train-test split identified 824 OTUs, whereas the 5-fold CV and LOO CV approaches revealed five OTUs consistently selected across all models (Supplementary Fig. 5a–c, Supplementary Data 2). This reduction underscores the benefit of cross-validated feature-selection models, which retain only features that are consistently informative across folds.

To evaluate the performance of machine learning models, we applied Wilcoxon rank-sum test and LEfSe^33^to identify features differentiating infection status and compared these results with the LOO-selected OTUs (Fig. 4c). Wilcoxon rank-sum test identified *Albugo* as the most significant OTU, and four of the five OTUs selected by the LOO-trained classifiers were also highlighted by these classical tests (Supplementary Fig. 5d, Supplementary Data 2), indicating agreement in detecting taxa with clear relative abundance differences.

In contrast, LEfSe^33^ did not identify any significantly differentially abundant OTUs using the default threshold (LDA score ≥ 2; Supplementary Data 2). However, when the threshold was relaxed, *Albugo* and four of the five OTUs selected by the LOO-trained classifiers were recovered, indicating that abundance differences detected by conventional methods were generally weak. These results show that while conventional methods highlight individual taxa with significant abundance differences, the machine learning classifiers identify a smaller set of OTUs that more effectively distinguish infection states more clearly.

**Fig. 5:**
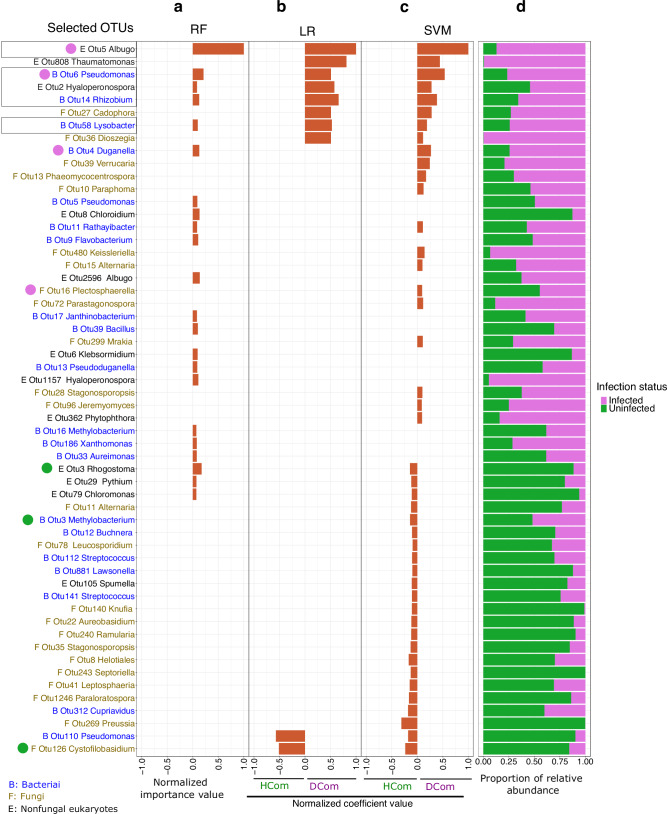
Health-associated and disease-associated microbial taxa identified via machine learning approaches. **a**–**c** Key microbial taxa identified based on feature importance in machine learning models (Random Forest [RF], Support Vector Machine [SVM], and Logistic Regression [LR]) (Supplementary Data 2). Histograms show normalized feature importance values: mean decrease in Gini impurity for RF, absolute coefficient values for SVM (linear kernel), and standardized coefficients for LR. For SVM and LR, negative values indicate OTUs enriched in uninfected leaves (health-associated microbes, HCom), while positive values indicate OTUs enriched in infected leaves (disease-associated microbes, DCom). **d** Bar plots depict aggregated relative abundances of selected OTUs in infected versus uninfected samples. Circles before OTU names indicate taxa selected for further experimental validation (green = HCom, purple = DCom). OTUs outlined with a border are those shared across all three models. Source data are provided as a Source Data file.

We also verified the taxonomic classification of OTUs identified through the LOO CV approach using BLAST (Supplementary Data 3). Unclassified OTUs with no BLAST hits were removed (*n* = 9), leaving 56 unique taxa for downstream analyses (Fig. 5). The SVM and LR models produced both positive and negative coefficients, enabling interpretation of microbial associations: negative scores indicated OTUs associated with uninfected plants—hereafter referred to as “health-associated” microbes (HCom)—whereas positive scores indicated OTUs enriched in infected plants, referred to as “disease-associated” microbes (DCom) (Fig. 5b, c). In contrast, RF provided only importance scores without directionality, requiring additional assessment to link OTUs to infection status (Fig. 5a).

Several studies have shown that plant disease induces the assembly of beneficial microbial consortiums that protect plants from infection^34,35^. Based on this concept, we propose that HCom and DCom play a role in modulating the infection of *Albugo*. The 56 key OTUs comprise 24 fungi, 20 bacteria, and 12 NFEuk. To experimentally validate their effects on *Albugo* infection, seven representative taxa were selected based on: (i) identification by at least two machine learning models, (ii) broad taxonomic diversity across kingdoms, and (iii) availability of isolates in culture collections. To link these isolates to the Machine Learning predicted OTUs, the full-length 16S rRNA or ITS sequences of our cultured isolates were aligned against the representative sequences of the key OTUs using BLASTn^36^, and isolates with the highest sequence identity (closest match) were selected. We chose *Pseudomonas viridiflava* (OTU6), *Plectosphaerella cucumerina* (OTU16), and *Duganella zoogloeoides* (OTU4) from DCom, and *Methylobacterium goesingense* (OTU3), *Cystofilobasidium macerans* (OTU126), *Rhogostoma epiphylla* sp. (OTU3), and *Sphingomonas melonis* (OTU15) from HCom. Given the known phenotypic variability between strains of the same microbial species, we acknowledge that marker gene sequences alone do not fully inform on the functional potential of the selected strains^37^. Nevertheless, this matching strategy allowed us to experimentally assess the potential roles of candidate microbes during *Albugo* infection.

### Health-associated community resulted in better biocontrol capacity compared to disease-associated community

**Fig. 6:**
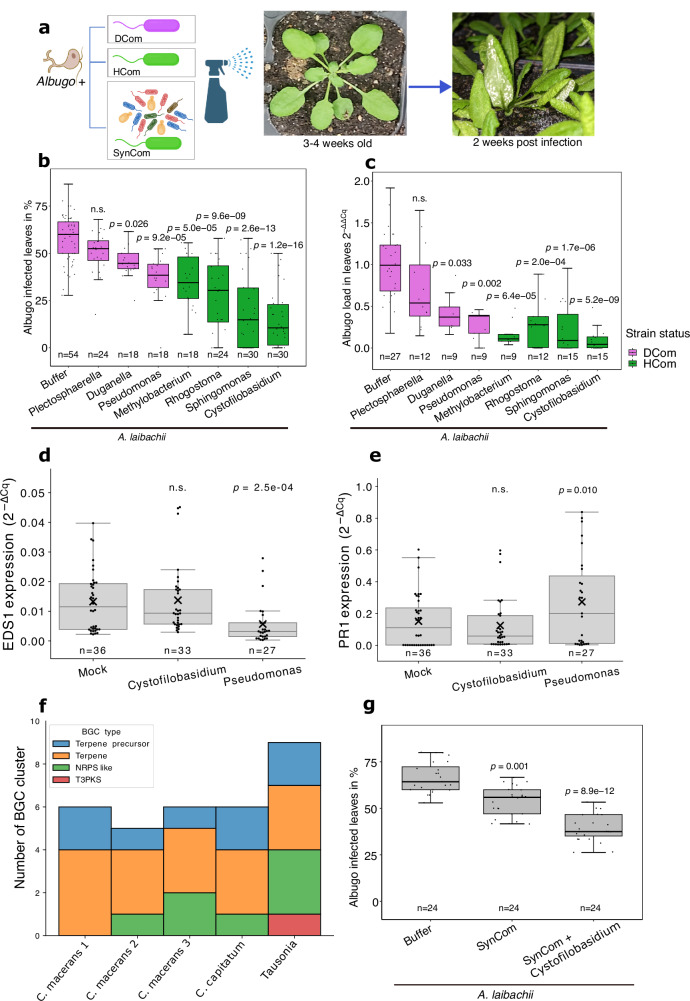
Validation of the biocontrol activity against *Albugo* infection using HCom and DCom strains. **a** Three- to four-week-old *Arabidopsis* plants were co-inoculated with *Albugo* and individual strains from health- or disease-associated taxa of interest (HCom and DCom taxa, respectively) (Fig. 5), under semi-sterile conditions. Part of the illustration is created in BioRender. Hu, Y. (2026) https://BioRender.com/wbmaigh. **b** Percentage of leaves infected with *Albugo* recorded two weeks post-inoculation. Colors indicate treatments with HCom or DCom taxa (*n* = 7). **c** *Albugo* load in leaves (*Albugo*:plant DNA ratio) estimated via qPCR on *Albugo*’s *EF1-α* and *A. thaliana*’s *EF1-α* (2^-ddCq^ method). **d**, **e** Relative expression levels of the plant defense gene EDS1 (**d**) and PR1 (**e**) in sterile *Arabidopsis* leaves measured by qPCR 24 h post-inoculation. *UBQ10* (At4g05320) was used as an internal reference. Exact *p* values from two-sided Tukey’s HSD tests (versus Mock) are shown for **d** and **e** (n.s., not significant). **f** Number of biosynthetic gene clusters (BGCs) and their annotated function from each yeast genome predicted by AntiSMASH^38^. The *Cystofilobasidium macerans* strain 3 is the OTU126 used in the infection assay with *Albugo*. **g** Plant-protecting effects of *Cystofilobasidium* within an *Arabidopsis* leaf SynCom. Percentage of leaves infected upon *Albugo* single inoculation, co-inoculation of *Albugo* with the 15-member SynCom, or co-inoculation of *Albugo* with the 15-member SynCom and *Cystofilobasidium* (*n* = 17). The n values are shown below each box. Exact *p* values from two-sided Dunn tests (versus the buffer control) are shown for (**b**), (**c**) and (**g**) (n.s., not significant). In all box plots, the center line denotes the median, box bounds the 25th and 75th percentiles, and whiskers the most extreme values within 1.5× the interquartile range; overlaid dots are individual samples and, in (**d**) and (**e**), the × marks the mean. The n values below each box indicate the number of biologically independent plants (**b**, **c**, **g**) or samples (**d**, **e**). Source data are provided as a Source Data file.

To investigate the protective effects of the selected HCom and DCom taxa against the infection caused by *Albugo*, first, a mixture of *Albugo* and each of the four microbes from HCom was sprayed onto *Arabidopsis* leaves (Fig. 6a). The level of protection was determined by measuring the percentage of infected leaves (Fig. 6b). All four candidates significantly decreased the infection caused by *Albugo* (Dunn test, *p* < 0.05). *Cystofilobasidium* exhibited the strongest effect, reducing *Albugo* levels by an average of 73%. *Sphingomonas* caused a 66% reduction, followed by *Rhogostoma* and *Methylobacterium*, which resulted in 53% and 40% decreases in *Albugo* infection, respectively (Fig. 6b). These observations were further confirmed by quantitative polymerase chain reaction (qPCR) analysis, which demonstrated that samples exposed to the HCom microbes exhibited substantially lower amounts of *Albugo* compared to control group (Dunn test, *p* < 0.05), with the average biomass of *Albugo* ranging from 72% to 90% (Fig. 6c). These results demonstrate that all the selected candidates associated with uninfected plants, namely, *Cystofilobasidium*, *Methylobacterium*, *Rhogostoma*, and *Sphingomonas*, significantly decreased the infection levels of *Albugo*. The *Methylobacterium*-treated plants exhibited the highest plant biomass, with an average of 0.96 grams fresh weight (Supplementary Fig. 6). Microscopy analysis revealed that *Rhogostoma* attached to the *Albugo* spores and feeds on free-living microbes in the environment (Supplementary Fig. 7, Supplementary Movie 1). These findings highlight the impact of HCom microbes in protecting against *Albugo* infection, with varying levels of effectiveness across different microbial taxa.

We then tested the effect of DCom microbes on the infection by *Albugo*. The *in planta* infection assay demonstrated that *Plectosphaerella* had no significant effect on the infection level of *Albugo* (Dunn test, *p* > 0.05). However, *Pseudomonas* and *Duganella* caused a decrease in infection of 36% and 20%, respectively (Dunn test, *p* < 0.05) (Fig. 6b). Likewise, qPCR outcomes supported the observed phenotype: *Plectosphaerella* did not significantly change *Albugo* biomass compared with the control, whereas *Pseudomonas* and *Duganella* caused a 72% and 58% reduction in *Albugo* biomass, respectively (Fig. 6c). The findings reveal that while some DCom microbes effectively reduce *Albugo* infection levels, their protective effects are lower than those of HCom microbes.

Since *Cystofilobasidium* exhibits promising biocontrol capacity against *Albugo* infection, we sought to explore the potential mechanism of *Cystofilobasidium-Albugo* interaction. We first performed a qPCR assay to assess if *Cystofilobasidium* induces plant defense. Spraying *Cystofilobasidium* on sterile *Arabidopsis* leaves does not increase the expression of two key defense marker genes EDS1 and PR1, whereas a known pathogen *Pseudomonas viridiflava* significantly altered their expression (Fig. 6d, e). This result suggests that *Cystofilobasidium* does not reduce *Albugo* colonization by directly inducing plant defense responses, but more likely through microbe-microbe interactions. To investigate potential mechanisms of microbial interaction between *Cystofilobasidium* and other microbes, we performed whole genome sequencing of four different strains of *Cystofilobasidium* and one isolate from genus *Tausonia*, a closely related genus of *Cystofilobasidium*. We achieved high quality genome assemblies to identify potential gene clusters responsible for the microbial interaction (Supplementary Table 3). We then predicted the potential biosynthetic gene clusters (BGC) of each genome with AntiSMASH^38^, and found that most of BGCs are related to terpene biosynthesis (Fig. 6f). While these genomic insights suggest that terpenoid compounds may be involved in the ecological fitness of *Cystofilobasidium*, their specific role in suppressing *Albugo* remains a preliminary hypothesis. These identified pathways provide a foundation for future functional studies to characterize the specific metabolites involved in this biocontrol activity.

We further evaluated the protective effects of *Cystofilobasidium* in a community context using a simplified synthetic microbial community (SynCom) consisting of 15 members (12 bacteria and 3 fungi; Supplementary Data 4). This SynCom was designed based on the *Arabidopsis* core microbiome, defined by the presence of OTUs in more than 95–98% of samples collected over three years of garden experiments (Supplementary Fig. 8a)^39^. It was previously shown to be stably reproduced both in vitro and *in planta*, displaying tolerance to metabolite perturbations^40^. In our current dataset, most OTUs corresponding to SynCom members were identified in the majority of samples (Supplementary Fig. 8b). Given its *in planta* stability and ecological relevance to our dataset, we employed this SynCom to test the effect of *Cystofilobasidium* in a community context. Application of the SynCom alone resulted in a moderate reduction (19.36%) in *Albugo* infection (Fig. 6g). The protective effect was further enhanced after adding the *Cystofilobasidium* OTU126 strain, resulting in a 40.25% reduction in infection levels (*p* < 0.05, Dunn test). This result confirms the ability of individual HCom members to contribute synergistically to community-level effects and emphasizes the biocontrol potential of the *Cystofilobasidium* strain.

## Discussion

In summary, we use *Albugo* infection as a natural model to examine how pathogen pressure reshapes the leaf microbiome and to identify microbial features linked to plant protection. By analyzing six years of multi-kingdom microbiome data from a natural *Arabidopsis thaliana* metapopulation, we characterize how infection interacts with host genotype, site, and microbial networks to structure community dynamics. Machine-learning models reveal a small set of microbial taxa strongly associated with infection status, and targeted isolation and experiments confirm that several of these candidates can suppress pathogen colonization, both alone and in a synthetic community. Our findings support the “cry-for-help” hypothesis^11^, providing a general framework for using long-term natural microbiome data to predict, test, and mechanistically characterize microbes with biocontrol potential (Supplementary Fig. 9).

The leaf endophytic microbial communities of wild *Arabidopsis thaliana* plants displayed spatial and compositional variability. Established factors such as sampling site, infection state, and plant genotype explained only part of the observed variability (Figs. 1 and 2), reflecting the multifactorial and dynamic nature of microbiome assembly^41^. Site effects were reported to be largely associated with local soil and climatic conditions in previous studies of *Arabidopsis* roots^6^ and crops such as sunflower and soybean^42^. Key climatic variables, including temperature^43^, solar radiation and humidity^9^, played important roles in structuring these communities. Furthermore, *Arabidopsis* microbiomes exhibited latitudinal gradients linked to drought adaptation and host genetic variation, reflecting the combined influence of environmental and genetic factors^8^. Beyond these drivers, natural populations frequently experience varying histories of prior or co-occurring infections; such unmeasured biotic interactions likely contribute to the remaining unexplained variability. Collectively, these findings highlight the context-dependent nature of plant microbiome assembly, shaped by dynamic interplay of environmental conditions, host genotype, and diverse biotic interactions.

Despite the influence of environment and host genotype, *Albugo* infection emerged as a key driver of leaf microbial diversity and network organization (Figs. 1 and 3). The observed reduction in microbial diversity may destabilize community interactions, rendering the microbiome more susceptible to disturbance^44^. Concurrently, microbial co-abundance networks from infected plants displayed greater size and modularity—a pattern also reported in the soil and endophytic communities of tobacco in the presence of the pathogen *Ralstonia*^45^. While these patterns highlight the restructuring of the microbiome under infection, they remain correlative, and the biological mechanisms require further exploration. Previous studies suggest that *Albugo* likely mediates these microbiome shifts through partial suppression of host immunity^46^, reprogramming of the leaf metabolome^47^, and the secretion of antimicrobial compounds that selectively inhibit competitors^48^. Unraveling precisely how pathogens actively engineer the leaf micro-environment to facilitate colonization represents a critical frontier in plant microbiome research.

Our results indicate that machine learning approaches can predict plant infection status accurately from microbiome data and identify key microbial features associated with infection. This approach captures patterns emerging from coordinated shifts across many organisms, allowing us to detect community-level interactions and non-linear relationships among taxa that collectively distinguish samples. Consequently, it yields a more specific set of infection-associated OTUs while providing predictive power on new datasets, consistent with recent studies applying machine-learning frameworks to soil and plant microbiomes^20,49^. Our data also suggests that machine learning methods outperform conventional differential abundance methods such as LEfSe^33^ in detecting subtle, coordinated community-level shifts. However, given our relatively modest sample size (337 samples), we did not employ emerging deep learning architectures such as convolutional neural networks or transformers. These strategies generally require significantly larger datasets to achieve high predictive power^50^, making classical classifiers a more appropriate and interpretable choice for this study. Future studies with larger sample sizes could benefit from exploring deep learning frameworks for microbiome-based disease prediction.

We also demonstrated that the HCom cercozoan *Rhogostoma* suppressed infection, potentially by preying on *Albugo*-associated microbes (Supplementary Fig. 7) or could be directly consuming zoospores, consistent with its known microbivorous behavior^51,52^. Notably, the basidiomycete yeast *Cystofilobasidium* provided the strongest protection among HCom members (Fig. 6). This yeast markedly reduced *Albugo* colonization without inducing the plant defense markers *PR1* and *EDS1*, suggesting that its protective effect operates through microbe–microbe interactions rather than host immune activation. Genomic analyses revealed that *Cystofilobasidium* possesses biosynthetic gene clusters for terpene production. Although direct experimental evidence for their antimicrobial activity is currently lacking, these metabolites serve as potential candidates for mediating microbial competition and suppression. Future work involving metabolomics or gene knockout studies will be essential to confirm whether these specific terpenoids are the primary drivers of the observed plant protection.

Synthetic communities provide a powerful tool to bridge the gap between in vitro assays and complex field phenotypes^53^. We acknowledge that our 15-member SynCom is a reductionist model that does not fully capture the extensive taxonomic variation and complexity of the natural phyllosphere microbiome. However, by constructing the community based on the persistent core microbiome, we targeted taxa with high ecological stability—a strategy increasingly recognized for developing robust biocontrol solutions^54,55^. Consistent with the view that core communities can act as a barrier against pathogen invasion^54^, the SynCom alone yielded a moderate reduction in *Albugo* infection. The addition of *Cystofilobasidium* further enhanced this protective effect, demonstrating its synergistic contribution to community-level defense. Ultimately, this focus on the core microbiome addresses a major challenge in scalability: by selecting ubiquitous and adaptable taxa, we align with ecological frameworks for designing resilient microbiomes for sustainable agroecosystems^56^.

Overall, our findings highlight the potential of integrating machine learning with natural plant microbiome data to identify effective biocontrol agents informed by ecological principles. Harnessing such biocontrol strategies can enhance microbiome stability and functional efficacy. Ultimately, these insights pave the way for developing customized, field-ready biocontrol solutions tailored to specific crop genotypes, soil types, and climatic conditions—addressing major agricultural challenges.

## Methods

### Diversity analysis

The operational taxonomic unit (OTU) tables for bacteria, fungi, and nonfungal eukaryotes, along with the corresponding genotype clusters for each sample, were obtained from our previous study^9^. Here, we used only the endophytic dataset, which consists of 351 samples (97 samples infected by *Albugo* and 254 uninfected). Infection was defined by the presence of characteristic white blister symptoms on the leaves; genotype data were available for 96 samples. The OTU tables were modified for diversity analysis by excluding samples with fewer than 50 reads. Subsequently, OTU abundance tables were utilized to compute Shannon’s H’ diversity index using the “estimate_richness” function in the Phyloseq package^57^ for estimating alpha-diversity. To assess between-sample diversity, relative abundance OTU tables were computed (“decostand,” vegan package)^58^ and transformed using log10(x + 1) before calculating Bray-Curtis dissimilarities, which were then employed for non-metric multidimensional scaling ordination (NMDS) using the “ordinate” function in Phyloseq. Permutational multivariate analysis of variance (PERMANOVA) based on Bray–Curtis dissimilarities was performed (“adonis2,“ vegan) to assess the effects of different factors on microbial community composition. Infection effects were tested separately within each year, site, and genotype, including only groups with ≥3 replicates per infection status. R² values represent the variance explained by each factor, with significance assessed using 10,000 permutations. For these stratified comparisons, weighted R² values were calculated from significant models (*p* < 0.05), with weights proportional to the number of samples in each subset. Site and genotype effects were also tested independently, and a full factorial “site × *g*enotype” model was applied to sites containing at least two genotypes with ≥3 replicates each. The means were compared using the nonparametric multivariate test for multiple group comparisons (“dunnTest,” FSA)^59^, with Benjamini-Hochberg adjusted *p*-values < 0.05), and the nonparametric ranked test for two groups (“wilcox.test,” stats package)^60^, *p* < 0.05). All analyses were conducted in R (version 4.1.2)^61^. All the scripts used for diversity analyses are available in the analysis_scripts/2_Diversity folder of the Git, and the cleaned OTU tables are provided in the preprocessed_tables/.

### Machine learning analysis

The OTU tables of bacteria, fungi, and nonfungal eukaryotes were independently converted to relative abundance within each microbial kingdom and subsequently merged into a single composite table. This ensured that cross-kingdom differences in sequencing depth and amplification efficiency did not bias the feature selection. OTUs present in fewer than five samples were removed, resulting in 2,543 OTUs across 337 samples. Each plant was labeled as infected or uninfected based on the presence of white rust caused by *Albugo*.

Machine learning analyses were performed in Python (version 3.7) using the scikit-learn package (version 0.22.2.post1)^62^. Four classifiers were applied: Random Forest (RF, RandomForestClassifier), Support Vector Machine (SVM, SVC with linear kernel), Logistic Regression (LR, LogisticRegression), and Multi-Layer Perceptron (MLP, MLPClassifier).

Prior to the three evaluation approaches, hyperparameters were optimized using a nested cross-validation framework, enabling simultaneous hyperparameter tuning and preventing data leakage to ensure unbiased model evaluation. This framework comprises two levels: an outer cross-validation loop used to estimate model performance, and an inner cross-validation loop used to select optimal hyperparameters. In each outer fold, the training data were further split within the inner loop to identify the best hyperparameters, after which the optimized model was evaluated on the held-out outer test fold. The outer loop employed stratified 5-fold cross-validation (StratifiedKFold, n_splits = 5, shuffle = True, random_state = 42), while the inner loop applied GridSearchCV with stratified 5-fold cross-validation using the macro-averaged F1-score as the optimization metric. The following hyperparameter grids were explored: SVM (C = [0.01, 0.1, 1, 10], linear kernel), logistic regression (C = [0.01, 0.1, 1, 10]), random forest (n_estimators = [50, 100, 200, 500, 1000], max_depth = [None, 10, 20]), and multilayer perceptron (hidden_layer_sizes = [(20,), (50,), (100,), (50, 20)], activation = [relu, tanh], alpha = [1e − 4, 1e − 3, 1e − 2, 1e − 1], learning_rate_init = [1e − 4, 1e − 3]). For each outer fold, the best-performing hyperparameters from the inner loop were used to train the model and generate predictions on the corresponding test fold. Final model configurations were determined based on the most frequently selected hyperparameters across outer folds (Supplementary Table 2): logistic regression (C = 10), random forest (n_estimators = 50, max_depth = 20), and multilayer perceptron (hidden_layer_sizes = (100,), activation = relu, alpha = 0.0001, learning_rate_init = 0.0001). For SVM, although C = 10 was selected more frequently across outer folds, C = 1 was chosen as it yielded superior performance in the later evaluations. All remaining parameters were kept at their default values in scikit-learn, and all classifiers used random_state = 42 to ensure reproducibility. These parameters were subsequently used in downstream analyses.

Three complementary approaches were applied to predict infection status, assess model robustness under different data partitioning strategies, and identify informative OTUs. First, a stratified 70:30 train–test split was performed, with models trained on 70% of the samples and evaluated on the remaining 30%. Feature (OTU) importance was derived from the training set using model coefficients for SVM and LR (model.coef_) and mean decrease in Gini impurity for RF (model.feature_importances_). Performance was assessed using accuracy, macro-averaged F1-score, precision, recall, and the area under the receiver operating characteristic curve (AUC).

Second, a combined feature selection and cross-validation approach was applied to assess model performance and identify informative OTUs. A stratified 70:30 train–test split was first performed. Recursive Feature Elimination with Cross-Validation (RFECV) was then applied on the training set only to select a subset of features that maximized cross-validation accuracy, thereby avoiding information leakage. Feature selection was conducted using stratified 5-fold cross-validation, in which model performance (accuracy) was evaluated iteratively across feature subsets. Model performance was subsequently evaluated using stratified 5-fold cross-validation on the training data for both full and reduced feature sets, and finally assessed on the independent test set. Performance metrics included accuracy, macro-averaged precision, recall, and F1-score, reported as mean ± standard deviation across folds. Because RFECV is not compatible with MLP models, the MLP classifier in the 5-fold CV analysis was evaluated without feature selection.

Third, a feature selection strategy based on leave-one-out (LOO) cross-validation was applied. Specifically, RFECV was performed on the full dataset using LOO as the internal cross-validation scheme to identify an optimal subset of features. Unlike the second approach, in which RFECV was applied strictly to the training set, this approach applied RFECV to the full dataset to maximize sample usage, with the primary aim of feature discovery, performance metrics serving as a secondary outcome. In this approach, each sample was sequentially left out during feature selection, and model performance (accuracy) was evaluated across all feature subsets and LOO splits. Following feature selection, the dataset was reduced to the selected OTUs, and model performance was evaluated using stratified 5-fold cross-validation. Performance metrics included accuracy, macro-averaged precision, recall, and F1-score, reported as mean ± standard deviation across folds. Feature rankings and corresponding model coefficients were extracted from the final RFECV model. Because RFECV is not compatible with multilayer perceptron models, this analysis was not performed for the MLP classifier. The OTUs selected and their corresponding importance values across all evaluation approaches are summarized in Supplementary Data 2. We then verified the taxonomic classification of OTUs identified through the LOO CV approach using BLAST. All the scripts used for machine learning analyses are available at the GitLab repository (https://gitlab.nfdi4plants.de/zmbp/MLAraHDCom/-/tree/main/analysis_scripts/4_Machinelearning?ref_type=heads).

### LEfSe and non-parametric test analysis

In addition to the machine learning models, we performed LEfSe^33^ analysis to identify OTUs differentially abundant between infected and uninfected samples and to estimate their effect sizes. LEfSe combines non-parametric statistical testing with linear discriminant analysis (LDA), making it suitable for detecting biologically meaningful differences in complex microbial communities^33^. The OTU table used for the machine learning models was reformatted using “lefse_format_input.py” and analyzed with “lefse_run.py.” We applied a reduced LDA score threshold of 0.001 (the default is 2.0), as no OTUs exceeded the default cutoff, and retained OTUs with adjusted *p*-value < 0.05 and LDA > 0.001. We also compared differences in OTUs between infected and uninfected samples using an independent non-parametric approach, using the “pairwise.wilcox.test“^60^ function in R^61^. *P*-values were adjusted using the Benjamini–Hochberg (BH) method, with significance defined as adjusted *p* < 0.05. Because LEfSe also incorporates non-parametric testing, both approaches identified the same set of OTUs that differed between infected and uninfected samples (Supplementary Data 2). Scripts used for this step are available at the GitLab repository (https://gitlab.nfdi4plants.de/zmbp/MLAraHDCom/-/tree/main/analysis_scripts/4_Machinelearning/ml_code/lefse_analysis?ref_type=heads).

### Microbial network analysis and properties

To construct microbial co-abundance networks, samples of infected and uninfected plants were separated, and the OTU tables of bacteria, fungi, and nonfungal eukaryotes were merged. The OTU tables were then filtered to retain only OTUs present in at least five samples, resulting in 2543 OTUs across 242 samples for uninfected plants and 1058 OTUs across 95 samples for infected plants. The filtered OTU tables were used to calculate correlations using the SparCC algorithm^63^, which employs Aitchison’s log-ratio framework and is designed for sparse compositional data. SparCC correlations were computed via the FastSpar platform^64^. Permuted p-values were generated from 1000 bootstrap datasets created by resampling the original count data. Correlations were recalculated for each bootstrap, and the observed correlations were compared to the distribution of bootstrap-derived correlations. The proportion of bootstrap correlations whose absolute value was equal to or greater than that of the observed correlation was taken as the permuted p-value. For downstream analyses, correlations with |*r* | ≥ 0.2 and *p* ≤ 0.01 were retained (Supplementary Data 5). Prior to selecting thresholds, modularity, number of nodes, and number of edges were plotted across different *p*-values and correlation thresholds to ensure an appropriate balance between network robustness and information content (Supplementary Fig. 2). Modularity analysis was performed using Python’s networkx package (version 3.1)^27^, and community detection was applied using the Louvain algorithm (“community_louvain.best_partition”)^65^. The modularity score for the detected modules was calculated using “community_louvain.modularity.” For network visualization and additional metrics, Cytoscape (version 3.7.1)^66^ was used to calculate node degree, closeness centrality, and network visualization. All the scripts used for network analysis are available at the GitLab repository (https://gitlab.nfdi4plants.de/zmbp/MLAraHDCom/-/tree/main/analysis_scripts/3_Network?ref_type=heads).

### Assembly of strains

Bacterial and fungal strains used in this study were isolated from *Arabidopsis* leaves collected from natural *Arabidopsis* populations^9^. Briefly, *Arabidopsis* leaves were processed to isolate epiphytic and endophytic microbes. Isolation was performed using different growth media, including NBA, TSB, R2A, and King’s B for bacterial strains and PDA and malt agar for fungal strains, and incubated at 16 °C, 22 °C, and 28 °C for 1 week. The isolated strains were purified by repeated streaking (2–3 times) to ensure purity, followed by subculturing and verification through 16S rRNA gene sequencing for bacteria and ITS gene sequencing for fungi. To identify isolates corresponding to the key features prioritized by the machine learning models, we aligned the full-length isolate sequences against the representative sequences of the target OTUs using BLASTn. Isolates exhibiting the highest sequence identity to the key OTUs were selected as the closest representatives. Purified strains were preserved in 30% glycerol stocks at −80 °C. Strains were selected for experimental setups based on machine-learning–derived importance scores distinguishing infected from uninfected plants (Fig. 5). From HCom, two bacterial strains and one yeast were selected, while from DCom, two bacterial strains and one fungal strain were included. Additionally, an HCom member, *Rhogostoma* (ID CCAP 1966/12), was obtained from the Culture Collection of Algae and Protozoa (CCAP, Scotland, United Kingdom).

SynCom members consisted of 12 bacterial strains and three yeasts; this composition was selected to reflect the natural dominance of bacterial diversity and abundance over fungi in the *Arabidopsis* phyllosphere, ensuring the SynCom mirrors the kingdom-level balance observed in the field^9,40^. Selected bacterial strains were activated on NBA and/or King’s B medium, while fungal strains were cultured on PDA at 22 °C for 48 h. Representative cultures of *Plectosphaerella*, HCom and DCom strains are shown in Supplementary Fig. 10, and representative cultures and the phylogenetic relationship of SynCom strains are shown in Supplementary Fig. 11. Notably, due to its slow growth rate and unique nutrient requirements, the *Methylobacterium* strain required specific growth conditions. It was cultivated on NBA supplemented with 1% methanol, added after autoclaving to ensure optimal growth. Similarly, *Rhogostoma* required a different approach and was cultured on NCL:PJ medium prepared from Prescott’s and James’s stock solutions (Supplementary Table 4). Representative patches formed by *Rhogostoma epiphylla* on NCL:PJ liquid culture are shown in Supplementary Fig. 12. Additionally, *Albugo*, being an obligate biotroph, required specialized growth conditions for its cultivation. The sequences of all strains are provided in Supplementary Data 3, while their growth conditions are detailed in Supplementary Method 1.

### Phylogenetic tree of SynCom strains and their establishment *in planta*

The assembled SynCom taxa represent a diverse set of dominant phyla, including *Actinomycetota, Pseudomonadota, Bacillota, Bacteroidota*, and *Basidiomycota*. The phylogenetic tree, constructed from 16S rRNA and ITS gene sequences (Supplementary Data 4), was generated by first checking sequence orientation using MAFFT with the ‘adjustdirection’ option^67^. Multiple sequence alignment was then performed in MEGA X^68^ using MUSCLE with default settings and up to 100 iterations. The tree was constructed using the Maximum Likelihood method in MEGA X, and visualized with iTOL^69^. Bootstrap values (100 replicates) are shown at the nodes as measures of branch support. To represent the occurrence of SynCom members in our dataset, we performed BLASTn^36^alignments between SynCom 16S rRNA/ITS sequences and representative OTU sequences from this study (Supplementary Data 4). OTUs with sequence identity ≥90% were considered matches. For each SynCom member, their occurrence across samples was calculated as the percentage of samples containing at least one of the matched OTUs. Materials for this analysis are available at the GitLab repository (https://gitlab.nfdi4plants.de/zmbp/MLAraHDCom/-/tree/main/analysis_scripts/6_Syncom_tree?ref_type=heads).

### Infections of *A. thaliana* leaves by HCom and DCom strains and quantification of *Albugo* biomass by qPCR

Overnight liquid cultures of bacteria and yeast were diluted in fresh medium to an OD_600_ of 0.2. The cultures were centrifuged at 1200 × *g* for 5 min, and the resulting pellets were resuspended in MgCl_2_ containing 2 µL of Silwet L-77. Spore and cell suspensions with concentrations of 25×10^4^ spores/mL or cells/mL were prepared for *Plectosphaerella*, *Albugo*, and *Rhogostoma* (Supplementary Method 1). Approximately 4–5 mL of each suspension was mixed with 5–6 mL of the *Albugo* solution and evenly sprayed onto 4–5-week-old *A. thaliana* seedlings (Ws-0 accessions grown on non-sterile soil) using airbrush guns. After spraying, the plants were covered with a light-transparent plastic bag and an additional black, lightproof plastic bag, then placed in a cold room at 8 °C for one day to promote *Albugo* colonization. Following this, the black plastic bags were removed, and the plants were stored in Sanyo cabinets under a short-day cycle of 10 h light at 22 °C and 14 h darkness at 16 °C. One day later, the light-transparent plastic bags were also removed. Two weeks post-inoculation, leaf disease symptoms were assessed by counting infected from uninfected leaves, quantified as a percentage, and fresh weights of plants were measured after removing the root. Leaves were then stored at −80 °C. DNA was extracted using the *FastDNA*^*TM*^ Spin Kit for Soil (MP Bio) following the manufacturer’s protocol. For qPCR, 15 µL reaction mixtures were prepared containing 7.5 µL of SYBR Green supermix, five µL of DNA (50 ng), 1.9 µL of nuclease-free water, and 0.3 µL each of forward and reverse primers (10 µL). Triplicate measurements were performed using a Bio-Rad CFX Connect real-time PCR detection system. The relative *Albugo* DNA biomass compared to plant DNA was calculated using the following oligonucleotide sequences: - *A. thaliana EF1-α*: 5’-AAGGAGGCTGCTGAGATGAA-3’, 5’-TGGTGGTCTCGAACTTCCAG-3’ - *Albugo*.

*EF1-α*: 5’-GTGTTCTGCACATCCACACC-3’, 5’-GACCTTGACGGATGAAAGGA-3’. dCq was calculated for each sample as Cq_*Albugo*_ – Cq_A.thaliana_. ddCq was then obtained by subtracting the average dCq of the *Albugo* control from each treatment dCq. Relative *Albugo* biomass was determined using 2^-ddCq^. Script for visualizing this data is available at the GitLab repository (https://gitlab.nfdi4plants.de/zmbp/MLAraHDCom/-/tree/main/analysis_scripts/5_InfectionAssay/scripts?ref_type=heads).

### The gnotobiotic microbial inoculation on *A. thaliana*

The gnotobiotic microbial inoculation system is adapted from Eitzen et al*.*^70^ and Ruhe et al*.*^46^ with some modifications. Briefly, *A. thaliana* Ws-0 (Wassilewskija) seeds were sterilized for 6–12 h with chlorine gas. Sterilized seeds were immediately sown on 0.5 x Murashige and Skoog (MS) medium with 0.75% agar and incubated under 8–16-h light-dark cycles with a 20 °C day and 16 °C night temperature with 60% humidity. After 7 days, the germinated seedlings were placed onto 12-well plates (Greiner bio-one) with each well filled with 2.5 mL 0.5 x MS-agar. Plates with seedlings were further incubated for 3 weeks before spraying. To spray *Pseudomonas viridiflava* and *Cystofilobasidium macerans*, we directly streaked fresh bacteria or yeast colonies ( < 5-day growth) from agar plates and resuspended them in 10 mM MgCl_2_ solution. We then diluted the strain resuspension to reach optical density of 600 nm (OD_600_) at 0.02 for *P. viridiflava* and 0.2 for *C. macerans*. After dilution, all microbial solutions were transferred into the airbrush guns (Conrad electronics, Hirschau, Germany) and sprayed two times ( ~ 40 μL each) with the air pump (Conrad electronics) at 0.5 bar pressure. After spraying, all 12-well plates were sealed with breathable tape and incubated under 8–16-h light-dark cycles with a 20 °C day and 16 °C night temperature with 60% humidity for 24 h before harvesting. Three leaves from each plant were collected for RNA extraction.

### qRT-PCR assay to assess the defense gene expression from plants

The qRT-PCR assay is adapted from Stuttmann et al.^71^. Specifically, total RNA was extracted using RNeasy Plant Mini Kit (Qiagen) following manufacturer’s procedure. After quantification, we first performed DNase treatment with 10 *µ*g total RNA using the TURBO DNA-free kit (Thermo Fisher Scientific), followed by reverse transcriptase (RT) reaction with SuperScriptIV^™^ (Invitrogen) reverse transcriptase following the manufacturer’s procedure. 20 µL RT reactions were diluted 1:5 and 5 µL used for qPCR reactions on a Bio-Rad CFX Connect real-time PCR Detection System with Bio-Rad SsoAdvanced Universal SYBR Green Supermix. *UBQ10* (At4g05320) transcript levels were used as an internal reference in all samples (primers: 5’-AGATCCAGGACAAGGAGGTATTC-3’, 5’-CGCAGGACCAAGTGAAGAGTAG-3’). The transcript levels of *A. thaliana* EDS1 and PR1 gene were calculated using the following oligonucleotide sequences: EDS1: 5’-CGAAGACACAGGGCCGTA-3’, 5’-AAGCATGATCCGCACTCG-3’; PR1: 5’-TTCTTCCCTCGAAAGCTCAA-3’, 5’-AAGGCCCACCAGAGTGTATG-3’. Primer efficiencies were between 90% and 110% for all oligos, and data was analyzed using the formula 2^−ddCq^. Gene expression was evaluated in at least three independent experiments with similar results.

### Whole genome sequencing and genomic analysis

The genomes of the three *Cystofilobasidium macerans* strains, one *C. capitatum* and one *Tausonia pullulans* were sequenced and assembled. Single yeast colonies were first inoculated into 10 mL potato dextrose broth (PDB) and grown overnight at 22 °C with moderate shaking (180 rpm). The overnight culture was then transferred to 740 mL of PDB in a sterile Erlenmeyer flask and incubated overnight at 22 °C with shaking at 120 rpm. Cells were harvested by centrifugation at 4000 × *g* for 20 min. The pellet was transferred to a sterile mortar using a spatula and ground in the presence of 20 g of 0.1 mm zirconia beads. Liquid nitrogen was added intermittently during grinding to aid in cell disruption and preserve DNA integrity. We used Quick-DNA™ Fungal/Bacterial Miniprep Kit (Zymo Research) to extract high molecular weight DNA from the tissue following the manufacturer’s instructions. After the DNA extraction, we used the 0.8x volume of AMPure beads (Beckman Coulter) for clean-up and excluding short DNA fragments. The DNA was sent on dry ice to BMKGene (Biomarker Technologies, Münster, Germany) for PacBio sequencing. The sequencing of *Cystofilobasidium macerans* strain 3 (OTU126) was done using our in-house Nanopore sequencing system (Oxford Nanopore Technologies, ONT). Nanopore library was prepared according to Oxford Nanopore Technologies Native Barcoding Kit 24 V14 (SQK-NBD114.24) protocol. The sequencing was conducted with the nanopore R10 flowcell operated using MinKNOW (v23.11.5, ONT). The raw fast5 data was basecalled and adapters and barcodes were trimmed using the default Dorado basecaller (v0.9, ONT). The raw PacBio reads (.bam files) were converted to FASTA format with Samtools^72^. Both PacBio and Nanopore reads were assembled to draft genome using Canu^73^ (v2.2). The genome statistics were assessed using QUAST (v5.0)^74^ and BUSCO (v5.2)^75^ using the basidiomycota_odb10 database as reference. Results are summarised in Supplementary Table 3. We next used the funannotate pipeline^76^for gene prediction using the *Cryptococcus* neoformans proteome downloaded from UniProt^77^as reference. All assemblies and Augustus gene predictions are stored at the Gitlab (https://gitlab.nfdi4plants.de/zmbp/MLAraHDCom/-/tree/main/data/Yeast_Genomes?ref_type=heads). AntiSmash^38^ fungi web server (https://fungismash.secondarymetabolites.org) was used for predicting biosynthetic gene clusters. The script, containing detailed parameters for this step, is available at GitLab repository (https://gitlab.nfdi4plants.de/zmbp/MLAraHDCom/-/blob/main/data/Yeast_Genomes/genome_assembly.txt?ref_type=heads).

### Infections of *A. thaliana* leaves by SynCom and HCom strains

The preparation of SynCom followed the methods described by Höhn et al.^40^ with some modifications (Supplementary Method 1). SynCom microbes were cultured individually for 48 h at 22 °C with shaking at 180 rpm, washed three times with 10 mM MgCl_2_ (centrifuged at 5000 × *g* for 5 min), and resuspended in 10 mM MgCl_2_. Cell densities were adjusted to an OD_600_ of 1 in 10 mM MgCl_2_. For the initial SynCom treatments, including SynCom + *Cystofilobasidium* and SynCom + *Cystofilobasidium* + *Rhogostoma*, equal ratios of all microbial members were combined and sprayed onto 4-week-old seedlings using airbrush guns. After inoculation, plants were kept overnight in autoclaving bags. *Albugo* plant infection was carried out 5 days after microbial inoculation to allow for microbial community establishment on the leaf. The *Albugo* spore solution (25 × 10^4^ spores/mL) was prepared and evenly sprayed onto seedlings grown under non-sterile conditions. To promote *Albugo*’s establishment, plants were enclosed in autoclaving bags and kept overnight in a cold room at 8 °C. Following this, plants were maintained under short-day conditions (10 h of light at 21 °C and 14 h of darkness at 16 °C) for 7 days. Leaf disease symptoms were quantified as a percentage of infected leaves. Script for visualizing this data is available at the GitLab repository (https://gitlab.nfdi4plants.de/zmbp/MLAraHDCom/-/tree/main/analysis_scripts/5_InfectionAssay/scripts?ref_type=heads).

### Microscopical observations

A mixture of *Albugo* spores and *Rhogostoma* cells, each at a concentration of approximately 100,000 cells/mL, was prepared and placed in microscope slides or plastic Petri dish (see Supplementary Method 1). Observations were made under an epifluorescence Axiophot microscope (Zeiss, up to 64x magnification) and/or inverted microscope (Zeiss LSM880) 2-8 days after setting up the mixture.

### Reporting summary

Further information on research design is available in the Nature Portfolio Reporting Summary linked to this article.

## Supplementary information

Supplementary Information

Peer Review file

Description of Additional Supplementary Files

Supplementary Data 1

Supplementary Data 2

Supplementary Data 3

Supplementary Data 4

Supplementary Data 5

Supplementary Movie 1

Reporting Summary

## Source data

Source data

## Data Availability

Amplicon sequencing data are published by Mahmoudi et al.^9^ and are accessible via the NCBI BioProject PRJNA961058 or through the DataPLANT/FDAT repository [10.57754/FDAT.61ckt-vm178]. The raw PacBio and Nanopore sequence reads, genome assemblies and genome annotation files generated for the *Cystofilobasidium* and *Tausonia* isolates are deposited in DataPLANT/FDAT [10.57754/FDAT.3c6y3-97g78]. OTU tables and all intermediate data files—including a README documenting each step—are available at the GitLab [https://gitlab.nfdi4plants.de/zmbp/MLAraHDCom]. Source data are provided with this paper.

## Code availability

All custom code and analysis scripts used in this study, including a README documenting each step, are available at the GitLab repository [https://gitlab.nfdi4plants.de/zmbp/MLAraHDCom].
