# Supplementary material for: Machine learning reveals biocontrol agents shaping disease outcome in natural Arabidopsis populations
Source: Nat Commun. 2026 Jul 28;17:7570. doi: 10.1038/s41467-026-75789-w (PMC13415569; doi:10.1038/s41467-026-75789-w)
Supplement: Supplementary file 1 — Supplementary Information [file 41467_2026_75789_MOESM1_ESM.pdf]

Machine learning reveals biocontrol agents shaping disease outcome in natural *Arabidopsis* populations

Mahmoudi and Hu *et al.*

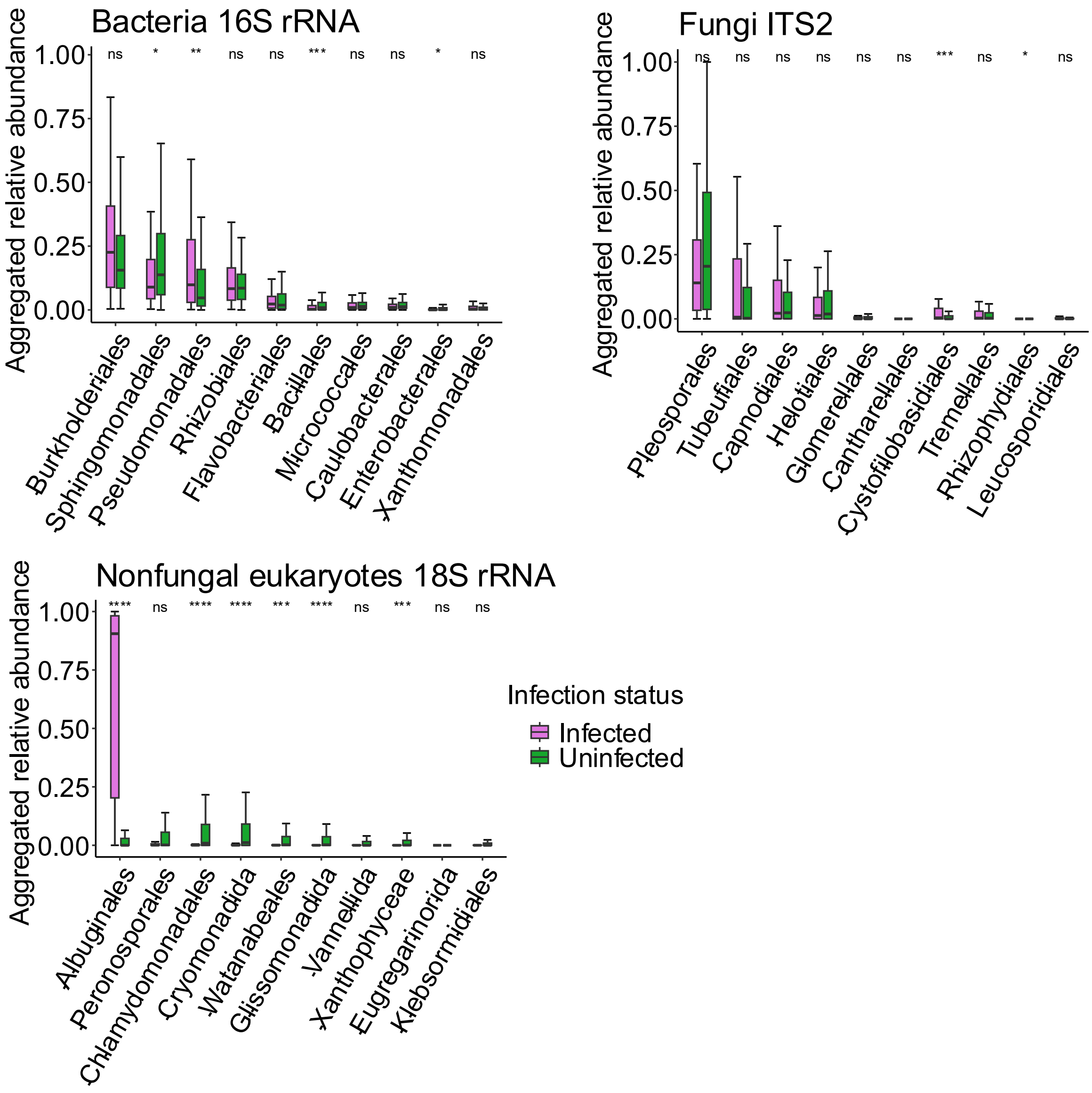

Supplementary Fig. 1. Changes in highly abundant microbial taxa at order level colonizing *A. thaliana*'s infected and uninfected leaves. Box plots (green = uninfected, purple = infected) show the relative abundance of the orders of bacteria, fungi and nonfungal eukaryotes in individual samples aggregated by infection status. Significance values between groups are based on Wilcoxon's test: n.s. ($p > 0.05$), * ($p \leq 0.05$), ** ($p \leq 0.01$), *** ($p \leq 0.001$), and **** ($p \leq 0.0001$). Source data are provided as a Source Data file.

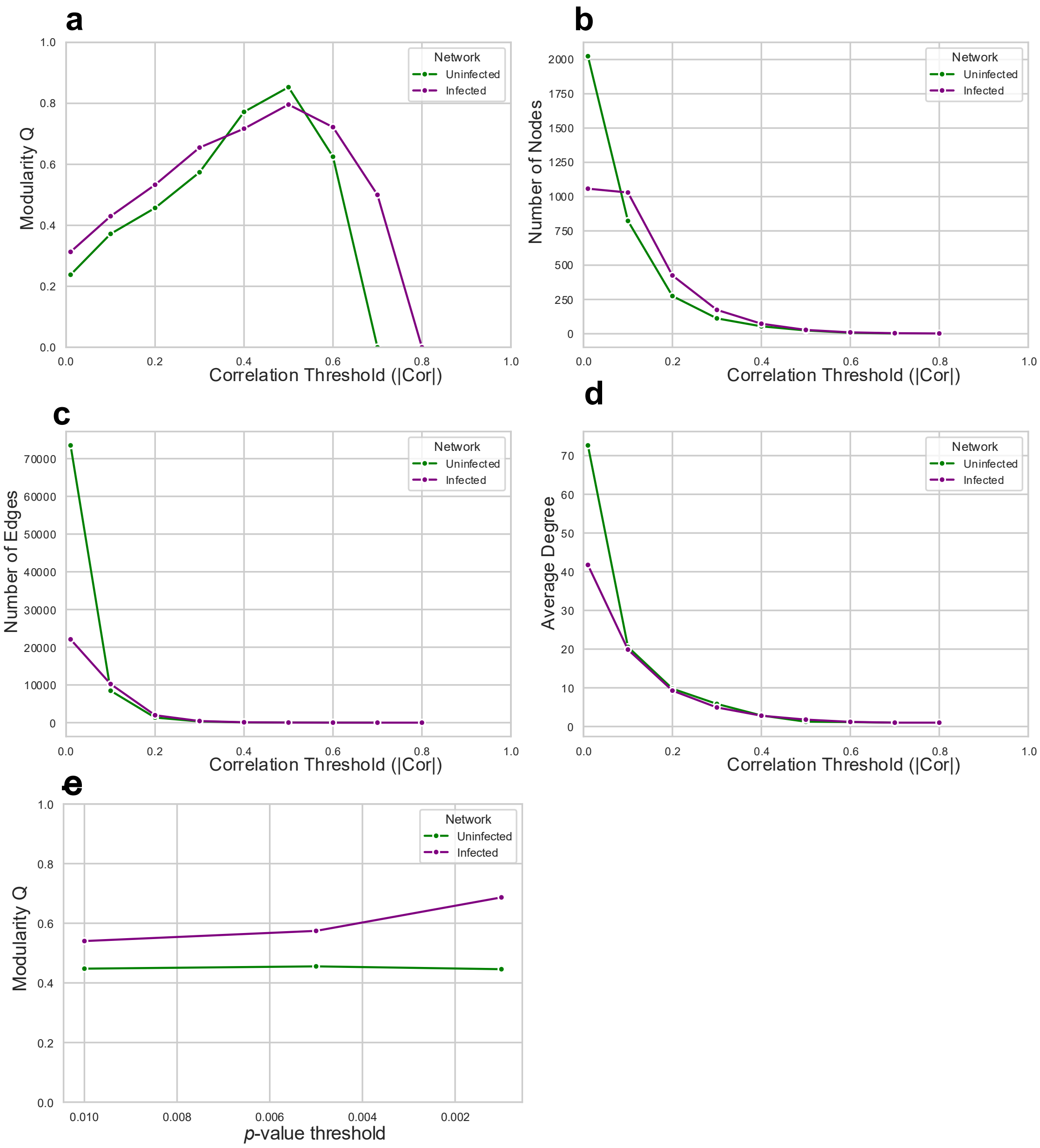

Supplementary Fig. 2. Exploration of correlation and *p*-value thresholds for network construction. (a–c) Line plots show changes in modularity (Q), number of nodes, and number of edges across different absolute correlation thresholds (|*r*|), calculated under $p \leq 0.01$. (**d**) Line plot shows average node degree across the same correlation thresholds. (**e**) Line plot shows modularity (Q) across different *p*-value thresholds, starting from 0.01. Green lines represent networks generated from uninfected samples, and purple lines represent networks generated from infected samples. Source data are provided as a Source Data file.

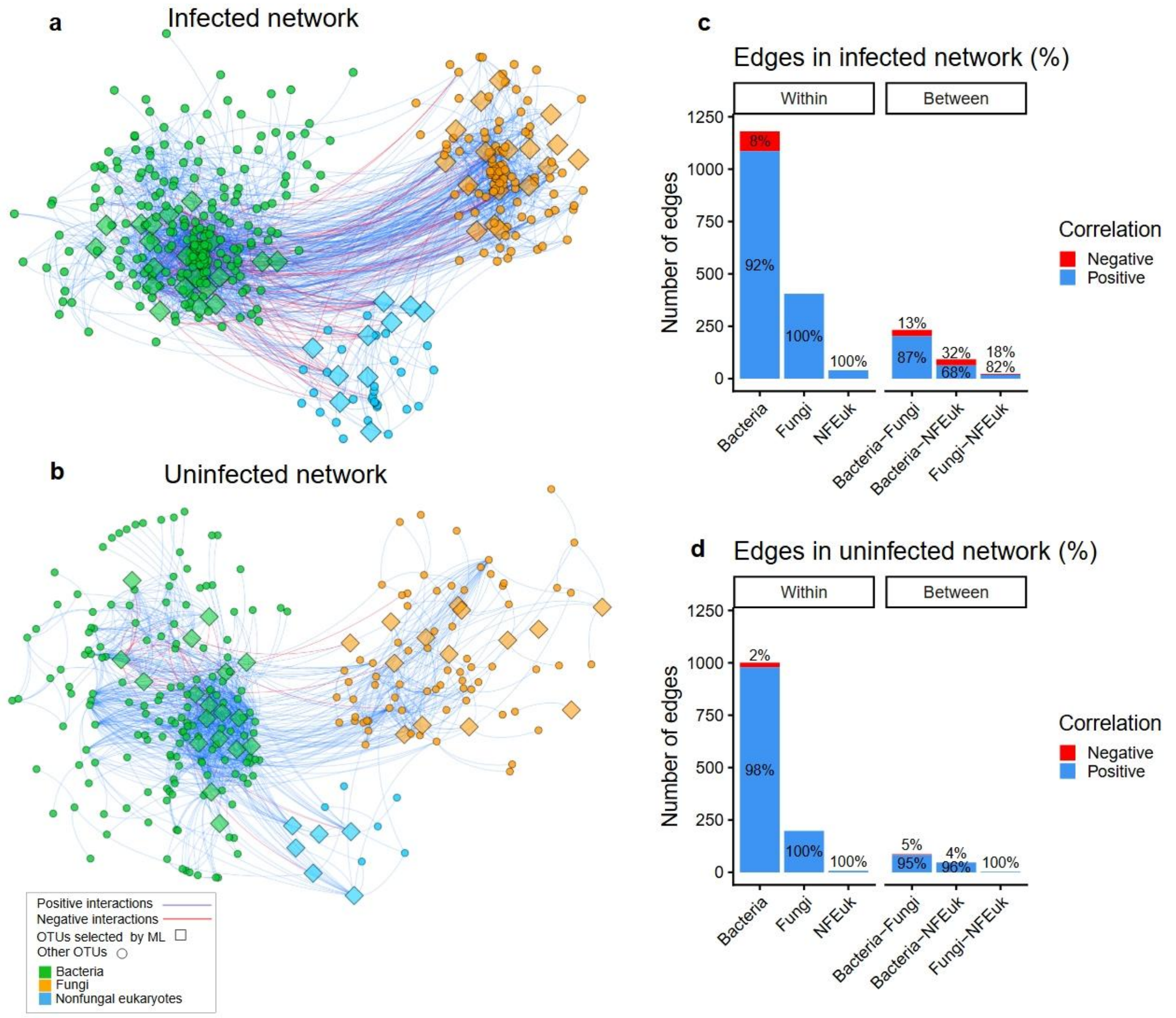

Supplementary Fig. 3. Multi-kingdom view of microbial co-abundance networks in infected and uninfected plants. Co-abundance networks are shown for infected (**a**) and uninfected (**b**). Nodes represent OTUs, and edges (connections between OTUs) indicate correlations. Nodes are color-coded by microbial taxa and grouped into microbial kingdoms (each circular cluster represents one kingdom). Microbes identified by machine learning analysis (Fig. 5) are highlighted with diamond-shaped nodes. Histograms show the number of positive and negative edges within and between kingdoms for (**c**) infected and (**d**) uninfected samples. Values on the bars indicate the proportion relative to the total count of edges represented by that bar. Source data are provided as a Source Data file.

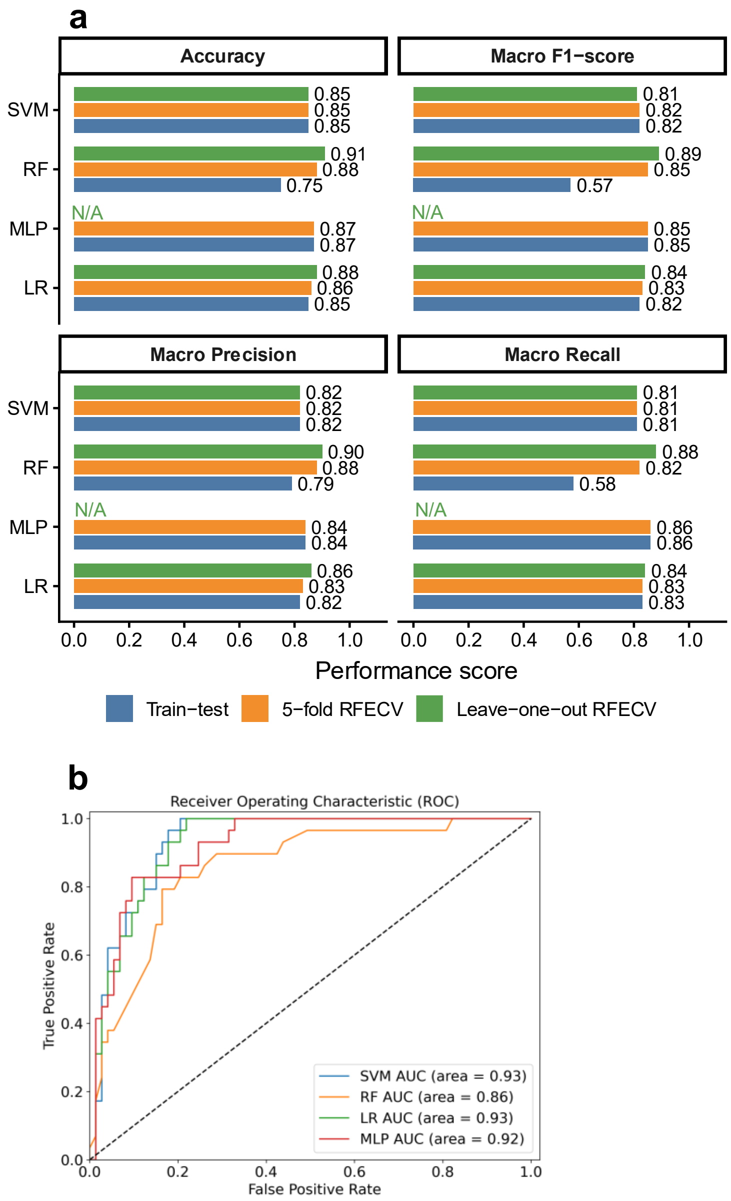

Supplementary Fig. 4. Comparative performance evaluation of classifiers across different validation strategies. (**a**) Performance metrics for each classifier across the three evaluation approaches. (**b**) Receiver operating characteristic (ROC) curves for each classifier on the test set (train-test split, Approach 1). "N/A" indicates values not applicable because RFECV is not compatible with the MLP model in the LOO CV approach; likewise, MLP results in the 5-fold CV were obtained without feature. Source data are provided as a Source Data file.

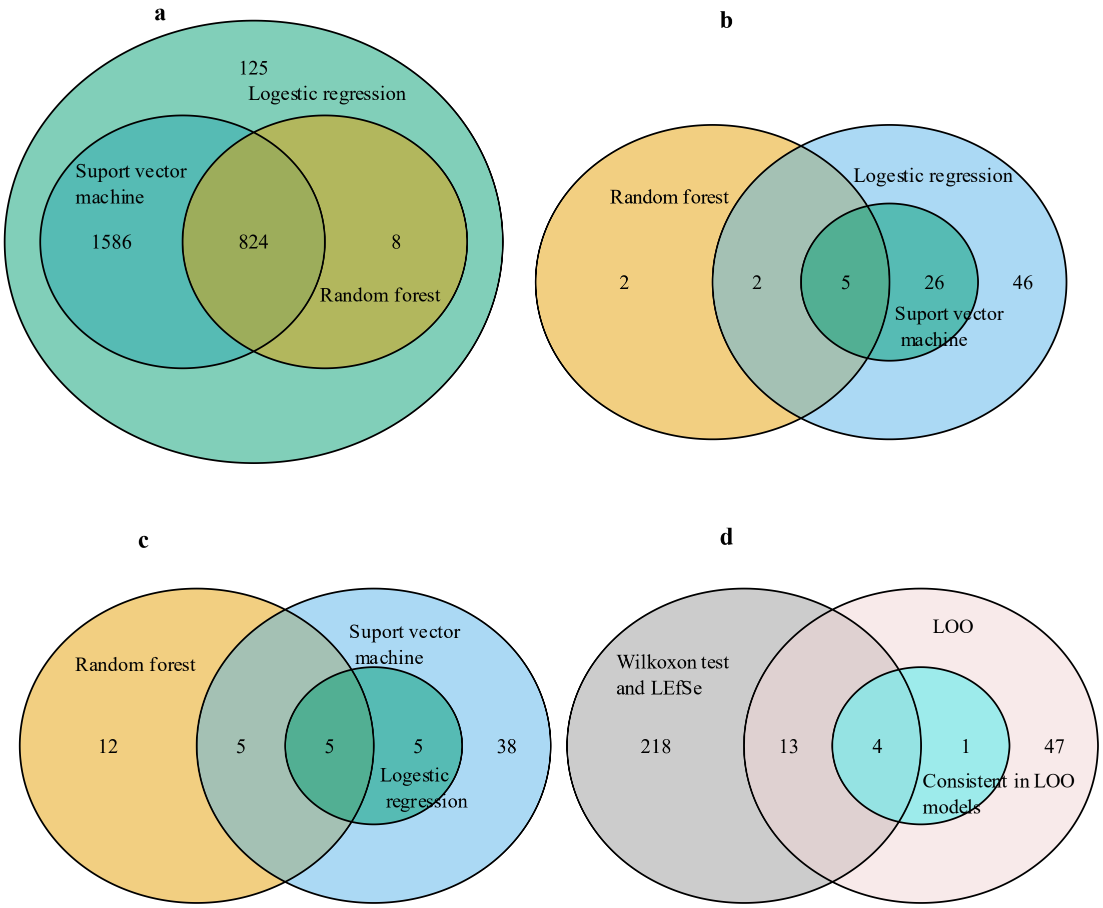

Supplementary Fig. 5. Comparison of important microbes for classifying uninfected and infected leaves using machine learning models. Four classification models were trained to distinguish infected from uninfected samples based on microbial signatures. (**a**) Venn diagram showing the number of common microbes with nonzero importance scores among different models during the 70% training phase (Train70). (**b**) Common important microbes identified by RFECV using 5-fold cross-validation. (**c**) Common important microbes identified by RFECV using leave-one-out (LOO) cross-validation. (**d**) Overlap of key microbes identified by RFECV LOO (combined from SVM, LR, and RF in panel **c**), Wilcoxon test and LEfSe analysis (LDA > 0.001 and adjusted $p < 0.05$). The small central circle represents the five OTUs shared from panel **c** (LOO). The complete list of microbes is provided in Supplementary Data 2. Source data are provided as a Source Data file.

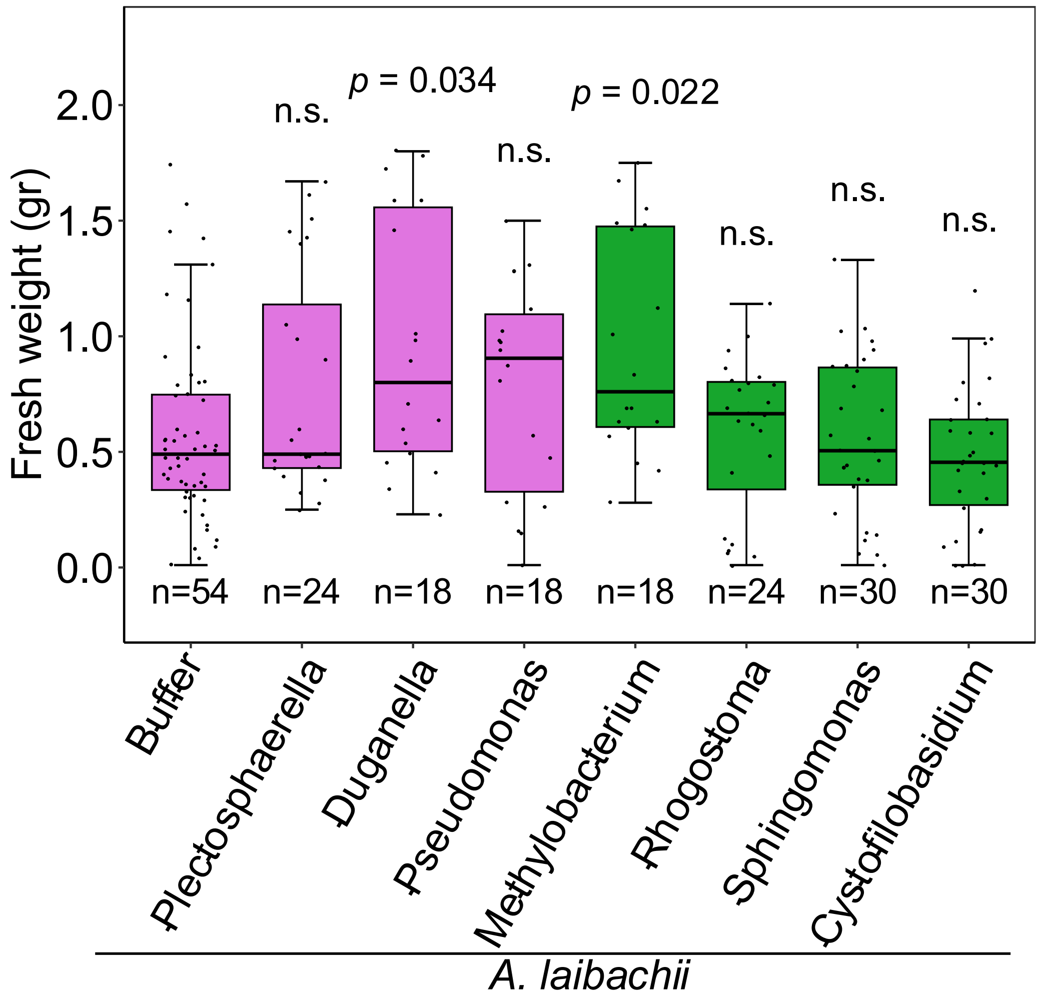

Supplementary Fig. 6. Fresh weight of plants inoculated with selected strains. Box plots showing the weights of leaves infected with Albugo in the presence of HCom strains (green) and DCom strains (purple). Exact p values from two-sided Tukey's HSD tests (versus the buffer control) are shown above each box (n.s., not significant). Individual plants are shown as dots, and the n values below each box indicate the number of biologically independent plants per group. In all box plots, the center line denotes the median, box bounds the 25th and 75th percentiles, and whiskers the most extreme values within 1.5× the interquartile range. Source data are provided as a Source Data file.

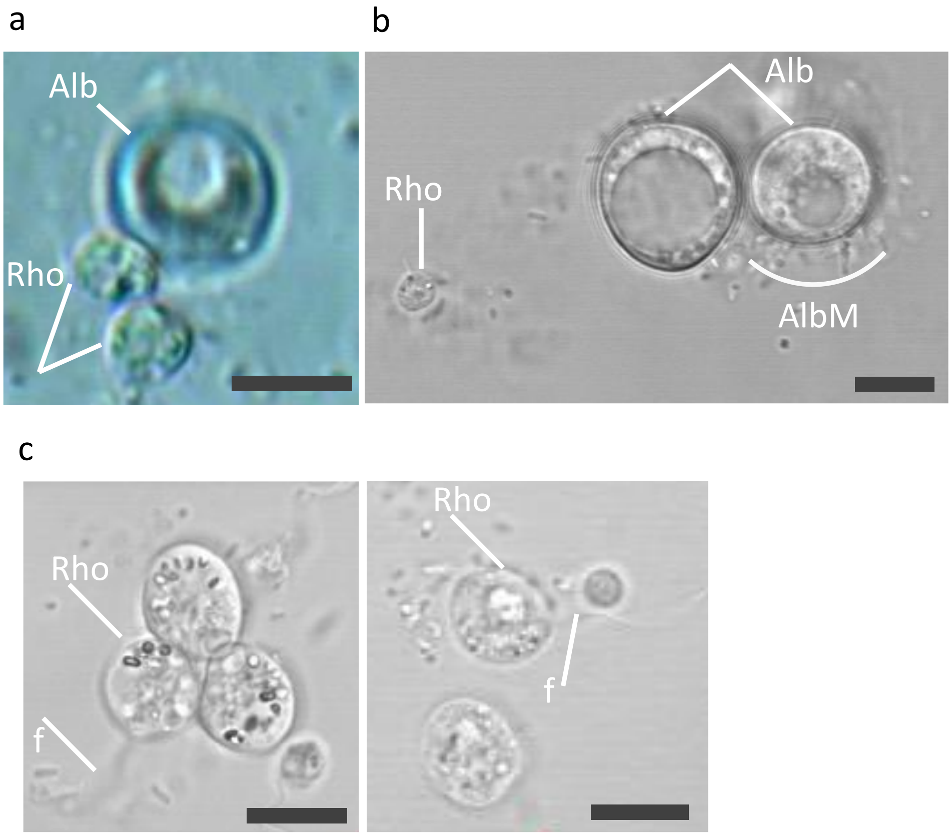

Supplementary Fig. 7. Possible interactions of *Rhogostoma epiphylla* with *Albugo*. (**a**) Single cells of *Rhogostoma* (Rho) attached to the spore of *Albugo* (Alb). (**b**) *Albugo* spores are associated with different microbes (AlbM). (**c**) *Rhogostoma* cells feeding on other microbes or *Albugo*'s zoospores via filopodia (f) (Supplementary Video1). Scale bar: 10µm.

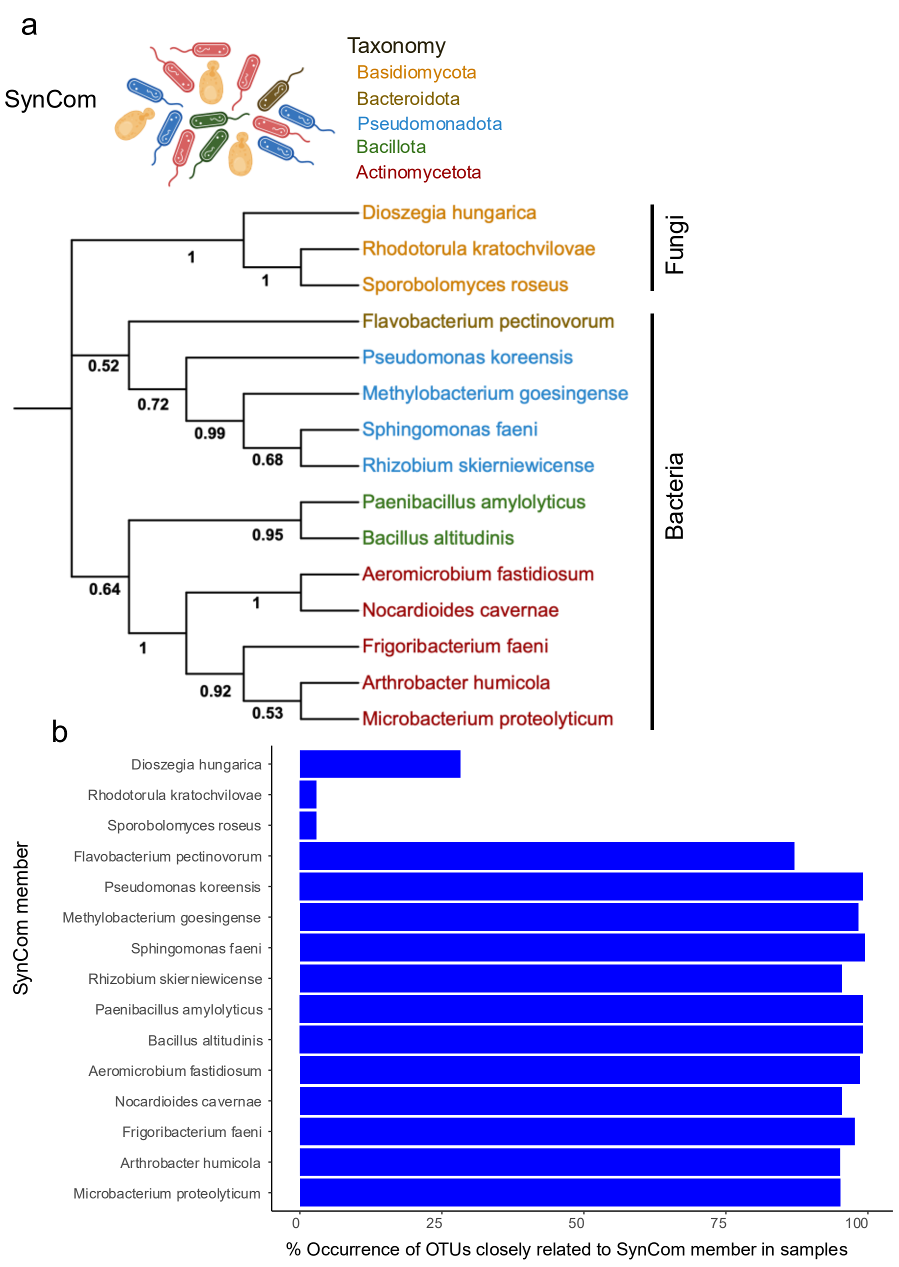

Supplementary Fig. 8. Phylogenetic tree of the SynCom strains and their relative abundance *in planta* and the field. (**a**) Phylogenetic tree of the SynCom strains (3 fungi and 12 bacteria) constructed from multiple sequence alignments of 16S rRNA and ITS gene sequences. Nodes are color-coded at the phylum level. Numbers at nodes indicate bootstrap support values. (b) The bar plots depict the occurrence of OTUs across samples, defined as the percentage of samples in which at least one OTU matched to a SynCom member based on BLASTn is present. Source data are provided as a Source Data file.

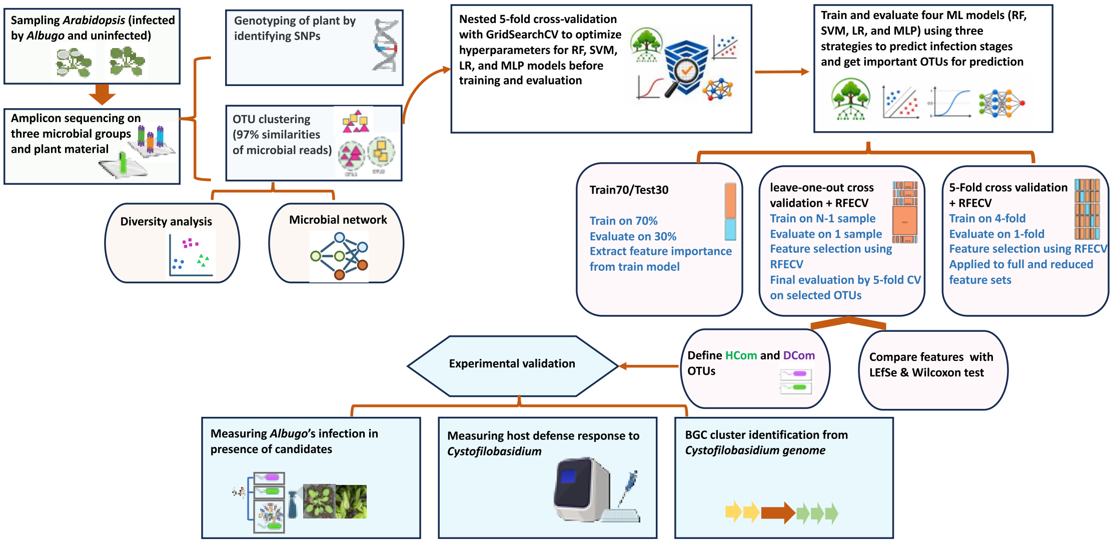

Supplementary Fig. 9. A schematic overview of the experimental design and analytical workflow of the study.

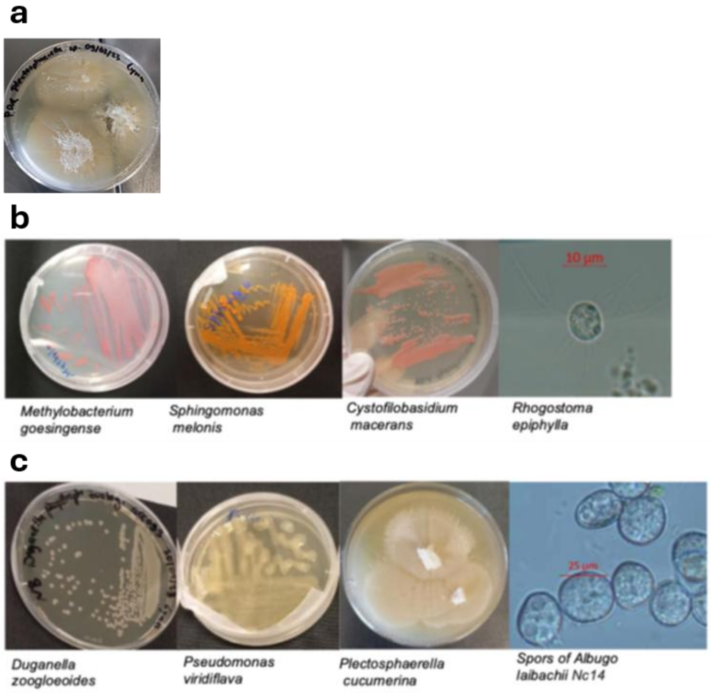

Supplementary Fig. 10. Culture of bacteria strains. (**a**) *Plectosphaerella cucumerina*, (**b**) HCom and (**c**) DCom strains.

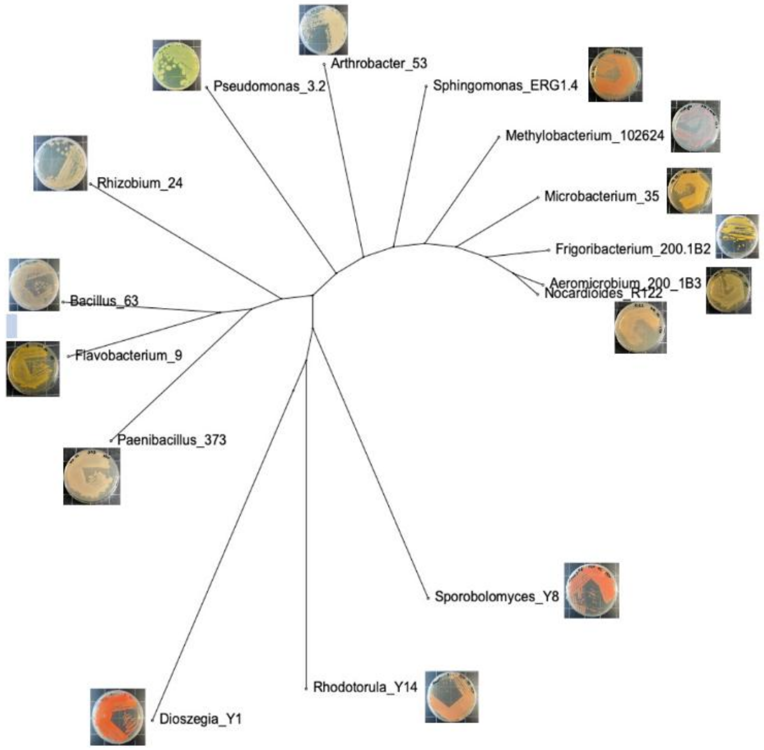

Supplementary Fig. 11. Cultures and phylogenetic relationship of SynCom strains.

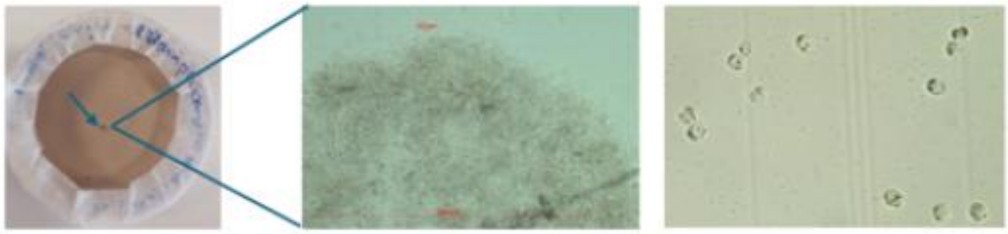

Supplementary Fig. 12. *Rhogostoma epiphylla* forms patches on the surface of NCL:PJ liquid culture.

Supplementary Table 1. Number of collected plant samples from natural ecosystems, their distribution across sampling sites over sampling years, assigned genotypes, and infection status (infected or uninfected). Numbers in parentheses indicate samples with genotype data.

Experiment	Time point	ERG		EY		JUG		PFN		WH		K69	
		Uninfected	Infected	Uninfected	Infected	Uninfected	Infected	Uninfected	Infected	Uninfected	Infected	Uninfected	Infected
Year1	Spring2014	2	2	2	4	3	3	3	0	3	3	0	0
Year2	Fall2014-Spring2015	7	7	5	7	6	7	6	0	6	6	3	0
Year3	Fall2015-Spring2016	9	3	6	6	12	0	11	0	6	6	10	0
Year4	Fall2016-Spring2017	9	3	11	0	10	4	7	0	7	7	12	0
Year5	Fall2017-Spring2018	6(6)	6(3)	9(8)	8(8)	11(8)	0	4(3)	0	7(3)	7(7)	9(9)	0
Year6	Fall2018-Spring2019	9(5)	0	10(7)	4(2)	10(8)	0	9(5)	0	10(7)	4(4)	4(3)	0

Total plant samples: 351.

Total plants with genotype data: 96.

Supplementary Table 2. Hyperparameters optimized using a nested 5-fold cross-validation framework. The table shows the evaluated parameters for each model across the five folds and the final hyperparameter values selected for downstream analysis.

Classifier	Parameter	Values tested	Fold 1	Fold 2	Fold 3	Fold 4	Fold 5	Final selected
SVM	C	0.01, 0.1, 1, 10	10	1	10	10	1	1
LR	C	0.01, 0.1, 1, 10	1	10	10	10	10	10
RF	n_estimators	50, 100, 200, 500, 1000	50	1000	100	50	50	50
RF	max_depth	None, 10, 20	NA	20	20	20	20	20
MLP	activation	relu, tanh	tanh	relu	relu	tanh	relu	relu
MLP	alpha	1e-4, 1e-3, 1e-2, 1e-1	0.01	0.1	1e-04	0.1	1e-04	0.1
MLP	hidden_layer_sizes	(20,), (50,), (100,), (50,20)	(100,)	(100,)	(50,)	(50,)	(100,)	(100,)
MLP	learning_rate_init	1e-4, 1e-3	1e-04	1e-04	1e-04	0.001	1e-04	1e-04

Supplementary Table 3. Genome assembly statistics for *Cystofilobasidium* and related *Tausonia* isolates.

**Assembly statistic**	**Yeast 1**	**Yeast 2**	**Yeast 3**	**Yeast 4**	**Yeast 5**
Species	*C. macerans*	*C. macerans*	*C. capitatum*	*C. macerans*	*T. pullulans*
Total assembly length (Mbp)	20.7	20.5	20.5	16.3	24
N50 contig (kbp)	1040.19	858.11	431.28	40.97	1235.02
Number of contigs	31	45	80	597	41
BUSCO completeness (%)	89.2	89.4	88.1	67.5	93
GC-content (%)	61.4	65.6	58.67	65.4	58.4
Percentage CDS (%)	61.96	66.3	64.34	65	56.18
Average gene size (bp)	2161.8	2230.1	2311.11	2076.77	2255.84
Protein-coding genes	7350	7411	7270	6255	7775

Supplementary Table 4. Composition of Prescott's & James's stock solutions (PJ).

**Stock solution no.**	**Ingredient**	**Amount per 100 mL**
**1**	$CaCl_2 \cdot 2H_2O$	0.43 g
	KCl	0.16 g
**2**	K_2HPO_4	0.51 g
**3**	$MgSO_4 \cdot 7H_2O$	0.28 g

Supplementary Method 1. Growth conditions and experimental preparation of all microbial strains

Bacterial isolates

The following bacterial species were cultured under the indicated conditions:

- **King's B medium (48 h):** *Sphingomonas melonis*
- **NBA supplemented with 1% methanol (48 h):** *Methylobacterium goesingense*
- **NBA (48 h):** *Duganella zoogloeoides*, *Pseudomonas viridiflava*, *Bacillus altitudinis*, *Rhizobium skierniewicense*, *Sphingomonas faeni*, *Pseudomonas koreensis*, *Flavobacterium pectinovorum*, *Microbacterium proteolyticum*, *Arthrobacter humicola*, *Frigoribacterium faeni*, *Aeromicrobium fastidiosum*, *Nocardioides cavernae*, and *Paenibacillus amylolyticus*.

Fungal isolates

The following fungal species were cultured under the indicated conditions:

- **PDA (48 h):** *Cystofilobasidium macerans*, *Dioszegia hungarica*, *Sporobolomyces roseus*, and *Rhodotorula kratochvilovae*.
- **PDA (2–4 weeks):** *Plectosphaerella cucumerina* (with some details coming later in the text).

Cultures of HCom, DCom and SynCom strains are shown in Supplementary Fig. 10 and 11.

Preparation of *Rhogostoma epiphylla* CCAP 1966/12

Rhogostoma epiphylla can grow in New Cereal Leaf – Prescott Liquid (NCL:PJ) medium which is provided by Culture Collection of Algae and Protozoa (CCAP, https://www.ccap.ac.uk).

Preparation of NCL:PJ Media

1. **Prepare stock solutions**
   Begin by preparing the stock solutions as detailed in Supplementary Table 4.
2. **Dilution of stock solutions**
   Take 1 mL of each PJ stock solution (1–3, as listed in Supplementary Table 4) and dissolve it in 1 L of deionized water.
3. **Heating and mixing**
   - Transfer 500 mL of the prepared solution into a microwave-safe container and heat until it begins to boil.
   - Add 1 g of wheatgrass powder to the boiling solution and boil again for approximately 5 minutes.
4. **Refilling volume**
   - Allow the solution to cool.
   - Refill the missing volume (due to condensation during boiling) with deionized water to bring the total back to 500 mL.

5. **Filtering and combining**
    - Filter the boiled solution through GF/C filter paper.
    - Combine the filtered solution with the remaining 500 mL of PJ solution.
6. **Final mixing and sterilization**
    - Mix the entire solution thoroughly.
    - Autoclave the mixture to sterilize it

Subculturing of *Rhogostoma epiphylla*

1. **Maintenance of cultures**
   Rhogostoma epiphylla was cultivated in NCL:PJ media. Initially, new subcultures were prepared by transferring 1 mL of an established *R. epiphylla* culture into a new Petri dish or a 50 mL culture flask filled to half the total volume with NCL:PJ media.
   Stock plates were transferred to growth chambers set to 16°C with an 8-hour light/16-hour dark cycle for long-term storage, covered with a sheet of paper to protect them from direct light. Older cultures were maintained by replenishing the media every 3-6 months (depends on the volume of media) weeks.
2. **Scaling up for *in planta* experiments**
   To obtain sufficient cell concentrations for *in planta* experiments, the following strategy was employed:
    - Prepare 10 mL cultures by mixing 1 mL of *R. epiphylla* culture taken from stock plates with 9 mL of NCL:PJ media.
    - Apply one of three treatments:
        1. Add 10 µL of Rifampicin to the culture.
        2. Add diluted *E. coli* strain DH5αpFru46 (grown on NBA media, diluted in 1 mL of nuclease-free water, and 200–500 µL taken).
        3. Combine *E. coli* and Rifampicin.
3. **Incubation**
   The cultures were stored in a paper box at 22°C for 5–7 days until they reached the desired cell concentration. The cell density was checked under a light microscope.
4. **Determination of cell concentration**
    - Take 15 µL of the culture suspension and load it onto a Neubauer counting chamber.
    - Observe under a light microscope (epifluorescence Axiophot microscope) (10–20× magnification) to count cells.
    - Calculate the concentration of cells (cells/mL) and adjust to approximately 25×10^6 cells/mL in a total volume of 10 mL.
    - Observation of patches
      During growth, undisturbed plates sometimes form patches on the liquid surface, visible to the naked eye. Microscopic examination of these patches revealed higher cell concentrations compared to liquid samples from the middle of the culture. These patches can be carefully harvested to obtain a concentrated suspension for experiments.

A typical patch is shown in Supplementary Fig. 12.

Preparation of *Albugo laibachii* Nc14 spores

Albugo laibachii Nc14, which had been maintained on *Arabidopsis thaliana* Ws-0 for over a year, was used in this experiment. Infected leaves were collected and placed in a 50 mL Falcon tube, filling approximately one-third to half of the tube with plant material. The tube was then filled with cold tap water.

The tube was placed on a rotator for 30–60 minutes to facilitate spore separation from the leaves. Subsequently, it was stored on ice for 1 hour. After this, the contents were vortexed and filtered into a new Falcon tube to remove debris.

To determine the concentration of *A. laibachii*, 15 μL of the prepared spore suspension was loaded onto a Neubauer counting chamber. Using a light microscope (epifluorescence Axiophot microscope), intact *A. laibachii* spores were counted, and the spore concentration (spores/mL) was calculated. The final concentration was adjusted to approximately 25×10^5 cell/mL in a total volume of 10 mL.

Preparation of *Plectosphaerella cucumerina* spores

1. **Culturing on PDA plates**
   Subculture *Plectosphaerella cucumerina* on Potato Dextrose Agar (PDA) plates. Incubate the plates at 22°C for approximately 3–4 weeks. During this period, the fungus will grow and produce hairy hyphae, indicating sufficient spore development (image below).
2. **Spore collection**
   - Collect sufficient fungal material from the PDA plate and transfer it to a 50 mL Falcon tube.
   - Add 25 mL of sterile distilled water (NFW). Note: Adjust the volume as needed, but ensure a final volume of 10 mL of spore suspension is prepared.
   - Vortex the tube thoroughly to dislodge and separate the spores into the water.
3. **Spore concentration determination**
   - Load 15 μL of the spore suspension onto a Neubauer counting chamber.
   - Use a light microscope (e.g., an epifluorescence Axiophot microscope) to count the spores.
   - Calculate the spore concentration in spores/mL based on the counts.
4. **Final adjustment**
   Adjust the final spore concentration to approximately 25×10^5 spores/mL in a total volume of 10 mL (Supplementary Fig. 10a).

A culture of *Plectosphaerella cucumerina* is shown in Supplementary Fig. 10a.
