# Supplementary material for: Machine learning reveals biocontrol agents shaping disease outcome in natural Arabidopsis populations
Source: Nat Commun. 2026 Jul 28;17:7570. doi: 10.1038/s41467-026-75789-w (PMC13415569; doi:10.1038/s41467-026-75789-w)
Supplement: Supplementary file 3 — Description of Additional Supplementary Files [file 41467_2026_75789_MOESM3_ESM.pdf]

Description of Additional Supplementary Files

File Name: Supplementary Data 1
Description: PERMANOVA results for the effects of infection, genotype and site on microbial variation. R^2 indicates the proportion of variance explained by each factor. Weighted R^2 values represent variance explained after weighting by sample size (SamplesFrom). Significant effects ($p < 0.05$) are indicated.

File Name: Supplementary Data 2
Description: Selected OTUs identified by different machine-learning models based on feature importance.
Column descriptions: Genus indicates the taxonomic assignment of each OTU. microbial_group denotes the higher-level microbial classification. OTUID corresponds to the unique OTU identifier. Feature_Importance represents the importance score assigned by the machine learning model for predicting sample groups. Model_Type indicates the type of machine learning model used. Data_Split_Method specifies the method used for training and cross-validation. Normalized_Feature_Importance shows the feature importance scaled relative to the most important OTU.

File Name: Supplementary Data 3
Description: OTUs selected for in planta experiments and their similarity to representative lab strains as determined by BLAST.
Column descriptions: OTUID indicates the unique identifier for each OTU. SelectedLabStrainsForEachOTU lists the lab strain used as the representative for that OTU in the experiment.
Performed in specifies the BLAST method used to determine similarity. 16S rRNA and ITS sequence specifies the type of sequence from the lab strain used for alignment.

File Name: Supplementary Data 4
Description: SynCom members selected for in planta experiments and their sequence information.
Column descriptions: Closest species indicates the taxonomic species most closely related to each SynCom member and their sequences.

File Name: Supplementary Data 5
Description: Correlation tables for infected and uninfected microbial association networks.

File Name: Supplementary Movie 1
Description: **Rhogostoma feeds with their filopodia in the solution containing Albugo spores.**
