# Supplementary material for: Machine learning reveals biocontrol agents shaping disease outcome in natural Arabidopsis populations
Source: Nat Commun. 2026 Jul 28;17:7570. doi: 10.1038/s41467-026-75789-w (PMC13415569; doi:10.1038/s41467-026-75789-w)
Supplement: Supplementary file 10 — Reporting Summary [file 41467_2026_75789_MOESM10_ESM.pdf]

nature portfolio

Corresponding author(s): Prof. Dr. Eric Kemen

Last updated by author(s): Jun 17, 2026

# Reporting Summary

Nature Portfolio wishes to improve the reproducibility of the work that we publish. This form provides structure for consistency and transparency in reporting. For further information on Nature Portfolio policies, see our Editorial Policies and the Editorial Policy Checklist.

## Statistics

For all statistical analyses, confirm that the following items are present in the figure legend, table legend, main text, or Methods section.

| n/a | Confirmed | |
|---|---|---|
| ☐ | ☒ | The exact sample size (*n*) for each experimental group/condition, given as a discrete number and unit of measurement |
| ☐ | ☒ | A statement on whether measurements were taken from distinct samples or whether the same sample was measured repeatedly |
| ☐ | ☒ | The statistical test(s) used AND whether they are one- or two-sided<br>*Only common tests should be described solely by name; describe more complex techniques in the Methods section.* |
| ☒ | ☐ | A description of all covariates tested |
| ☐ | ☒ | A description of any assumptions or corrections, such as tests of normality and adjustment for multiple comparisons |
| ☐ | ☒ | A full description of the statistical parameters including central tendency (e.g. means) or other basic estimates (e.g. regression coefficient) AND variation (e.g. standard deviation) or associated estimates of uncertainty (e.g. confidence intervals) |
| ☐ | ☒ | For null hypothesis testing, the test statistic (e.g. *F*, *t*, *r*) with confidence intervals, effect sizes, degrees of freedom and *P* value noted<br>*Give P values as exact values whenever suitable.* |
| ☒ | ☐ | For Bayesian analysis, information on the choice of priors and Markov chain Monte Carlo settings |
| ☒ | ☐ | For hierarchical and complex designs, identification of the appropriate level for tests and full reporting of outcomes |
| ☒ | ☐ | Estimates of effect sizes (e.g. Cohen's *d*, Pearson's *r*), indicating how they were calculated |

*Our web collection on statistics for biologists contains articles on many of the points above.*

## Software and code

Policy information about availability of computer code

| | |
|---|---|
| Data collection | Amplicon sequencing data for bacteria, fungi, and non-fungal eukaryotes, as well as host whole-genome sequencing data, were generated as described previously (Mahmoudi et al., 2024, https://doi.org/10.1093/ismeco/ycae103). Amplicon sequencing was performed using standard Illumina platforms following manufacturer protocols. Yeast whole-genome sequencing was performed using PacBio or Oxford Nanopore (ONT) long-read sequencing platforms. No custom software was developed specifically for data collection in this study. |
| Data analysis | Data analysis was performed using statistical, computational, and machine-learning methods. Alpha diversity (Shannon's H) was calculated with estimate_richness (Phyloseq), and Bray-Curtis dissimilarities on log10(x + 1)-transformed OTU tables were used for NMDS ordination (ordinate, Phyloseq). PERMANOVA (adonis2, vegan) assessed the effects of infection, site, and genotype. These analyses were conducted in R (version 4.1.2). Machine learning classifiers, including Random Forest, SVM, Logistic Regression, and MLP, were implemented in Python (version 3.7) using the scikit-learn (version 0.22.2.post1) package, with feature selection via RFECV for all but MLP, and model performance evaluated using accuracy, F1-score, precision, recall, and AUC. Differentially abundant OTUs were also identified using LEfSe and non-parametric tests. Microbial co-abundance networks were constructed using SparCC implemented in FastSpar, and community structure was analyzed with the Louvain algorithm in NetworkX; networks were visualized and metrics computed in Cytoscape. Phylogenetic trees of SynCom strains were generated using MAFFT, MUSCLE, and Maximum Likelihood in MEGA X, and visualized with iTOL.<br>Genome assembly of selected yeast strains was performed using Canu, and annotated using Funannotate. Schematic illustrations of figures were generated with BioRender. |

For manuscripts utilizing custom algorithms or software that are central to the research but not yet described in published literature, software must be made available to editors and reviewers. We strongly encourage code deposition in a community repository (e.g. GitHub). See the Nature Portfolio guidelines for submitting code & software for further information.

## Data

Policy information about availability of data

All manuscripts must include a data availability statement. This statement should provide the following information, where applicable:

- Accession codes, unique identifiers, or web links for publicly available datasets
- A description of any restrictions on data availability
- For clinical datasets or third party data, please ensure that the statement adheres to our policy

Amplicon sequencing data have been deposited in the NCBI Sequence Read Archive under BioProject accession number PRJNA961058. All scripts used for data processing, statistical analyses, machine-learning workflows, and network analyses, as well as processed OTU tables and adjacency matrices for networks, are available at https://gitlab.nfdi4plants.de/zmbp/MLAraHDCom. Source Data underlying the figures are provided with this paper.

## Research involving human participants, their data, or biological material

Policy information about studies with human participants or human data. See also policy information about sex, gender (identity/presentation), and sexual orientation and race, ethnicity and racism.

| | |
|---|---|
| Reporting on sex and gender | This study did not involve human participants, human data, or human biological material. Considerations of sex and gender are therefore not applicable. |
| Reporting on race, ethnicity, or other socially relevant groupings | This study did not involve human participants or human-derived data. Reporting on race, ethnicity, or other socially relevant groupings is therefore not applicable. |
| Population characteristics | This study did not involve human participants. Population characteristics are not applicable. |
| Recruitment | No human participants were recruited for this study. |
| Ethics oversight | Ethical approval was not required as this study did not involve human participants, human data, or human biological material. |

Note that full information on the approval of the study protocol must also be provided in the manuscript.

# Field-specific reporting

Please select the one below that is the best fit for your research. If you are not sure, read the appropriate sections before making your selection.

☐ Life sciences ☐ Behavioural & social sciences ☒ Ecological, evolutionary & environmental sciences

For a reference copy of the document with all sections, see nature.com/documents/nr-reporting-summary-flat.pdf

# Ecological, evolutionary & environmental sciences study design

All studies must disclose on these points even when the disclosure is negative.

| | |
|---|---|
| Study description | This study investigated how natural variation in the Arabidopsis thaliana leaf microbiome relates to infection by the obligate biotrophic pathogen Albugo laibachii, and whether microbiome signatures can be used to identify biocontrol agents. We analyzed multi-year, multi-site amplicon sequencing data from natural Arabidopsis populations, combined with host genotype information, microbial network analyses, and machine-learning–based classification. Selected microbial taxa were experimentally validated in controlled infection assays and synthetic community experiments. |
| Research sample | The research sample consisted of Arabidopsis thaliana plants collected from six natural populations near Tübingen, Germany, sampled annually over six years (2014–2019). Endophytic leaf microbiomes (bacteria, fungi, and non-fungal eukaryotes) were analyzed using amplicon sequencing. Host genotype information was available for a subset of samples. These samples were chosen to represent naturally occurring host–microbiome–pathogen interactions under field conditions. |
| Sampling strategy | Samples were collected annually from predefined field sites using a consistent sampling protocol described in ref. 9. Sample sizes were determined by field availability and population sizes rather than a priori power calculations, which is standard practice for ecological field studies. Statistical analyses appropriate for unbalanced designs were used, and multiple validation strategies were applied for machine-learning analyses. |
| Data collection | Leaf samples were collected in the field by trained researchers and processed under standardized conditions. Infection status was assessed visually based on characteristic Albugo symptoms. DNA extraction, different sequencing methods and host genotyping were performed using established protocols as described in the Methods and referenced literature. |
| Timing and spatial scale | Sampling was conducted annually between 2014 and 2019 across six field sites within a regional landscape near Tübingen, Germany. This temporal and spatial design captured natural interannual and site-dependent variation in microbiome composition and infection dynamics. |
| Data exclusions | OTUs with fewer than 50 sequencing reads in all the samples were excluded prior to analysis, as pre-defined in the data-processing pipeline. No other data were excluded. |

| | |
|---|---|
| Reproducibility | Key experimental findings were validated using independent biological replicates. Infection assays and qPCR analyses were repeated with consistent results. Computational analyses were performed using documented and version-controlled scripts, and machine-learning performance was assessed using multiple cross-validation strategies to ensure robustness. |
| Randomization | Randomization was not applicable for field sampling, as plants were sampled based on their natural occurrence. For controlled infection assays, plants were assigned to treatments without systematic bias. |
| Blinding | Blinding was not applied during data collection or analysis. Infection phenotypes were assessed using objective visual criteria and quantitative molecular measurements, and microbiome and machine-learning analyses were performed on coded datasets. |

Did the study involve field work? [x] Yes [ ] No

## Field work, collection and transport

| | |
|---|---|
| Field conditions | Field sampling was conducted under natural environmental conditions typical for temperate Central European habitats. No environmental parameters (e.g. temperature or rainfall) were experimentally manipulated during sampling. |
| Location | Samples were collected from six field sites located in the greater Tübingen region, Baden-Württemberg, Germany. Detailed coordinates of each site were recorded in Supplementary Table 1. |
| Access & import/export | All field sites were publicly accessible or sampled with permission of the respective landowners or managing authorities. Sampling involved non-protected plant species and did not require specific collection, import, or export permits under local or national regulations. No material was transported across international borders. |
| Disturbance | Field sampling caused minimal disturbance to the environment. Only small amounts of leaf material were collected from individual plants, and no long-term impact on plant populations or habitats was observed. |

# Reporting for specific materials, systems and methods

We require information from authors about some types of materials, experimental systems and methods used in many studies. Here, indicate whether each material, system or method listed is relevant to your study. If you are not sure if a list item applies to your research, read the appropriate section before selecting a response.

### Materials & experimental systems

| n/a | Involved in the study |
|---|---|
| [x] | [ ] Antibodies |
| [x] | [ ] Eukaryotic cell lines |
| [x] | [ ] Palaeontology and archaeology |
| [ ] | [x] Animals and other organisms |
| [x] | [ ] Clinical data |
| [x] | [ ] Dual use research of concern |
| [ ] | [x] Plants |

### Methods

| n/a | Involved in the study |
|---|---|
| [x] | [ ] ChIP-seq |
| [x] | [ ] Flow cytometry |
| [x] | [ ] MRI-based neuroimaging |

## Animals and other research organisms

Policy information about studies involving animals; ARRIVE guidelines recommended for reporting animal research, and Sex and Gender in Research

| | |
|---|---|
| Laboratory animals | This study did not involve laboratory animals. |
| Wild animals | This study did not involve wild animals. |
| Reporting on sex | This study did not involve animals for which sex is a relevant biological variable. Microorganisms and plants were studied. |
| Field-collected samples | This study did not involve field-collected animal samples. Field collection of plant material is described in the “Field work, collection and transport” section. |
| Ethics oversight | Ethical approval was not required as this study did not involve laboratory or wild animals. |

Note that full information on the approval of the study protocol must also be provided in the manuscript.

# Dual use research of concern

Policy information about dual use research of concern

## Hazards

Could the accidental, deliberate or reckless misuse of agents or technologies generated in the work, or the application of information presented in the manuscript, pose a threat to:

| No | Yes | |
|---|---|---|
| ☒ | ☐ | Public health |
| ☒ | ☐ | National security |
| ☒ | ☐ | Crops and/or livestock |
| ☒ | ☐ | Ecosystems |
| ☒ | ☐ | Any other significant area |

## Experiments of concern

Does the work involve any of these experiments of concern:

| No | Yes | |
|---|---|---|
| ☒ | ☐ | Demonstrate how to render a vaccine ineffective |
| ☒ | ☐ | Confer resistance to therapeutically useful antibiotics or antiviral agents |
| ☒ | ☐ | Enhance the virulence of a pathogen or render a nonpathogen virulent |
| ☒ | ☐ | Increase transmissibility of a pathogen |
| ☒ | ☐ | Alter the host range of a pathogen |
| ☒ | ☐ | Enable evasion of diagnostic/detection modalities |
| ☒ | ☐ | Enable the weaponization of a biological agent or toxin |
| ☒ | ☐ | Any other potentially harmful combination of experiments and agents |

# Plants

| | |
|---|---|
| Seed stocks | Arabidopsis thaliana ecotype Ws-0 (Wassilewskija, Russia) seeds were produced in greenhouse (ZMBP Tübingen Germany). |
| Novel plant genotypes | No novel plant genotypes were generated in this study. |
| Authentication | Plant species identity (Arabidopsis thaliana) was confirmed based on morphology and prior genomic characterization as described in the Methods. |
