# Supplementary material for: Machine learning reveals biocontrol agents shaping disease outcome in natural Arabidopsis populations
Source: Nat Commun. 2026 Jul 28;17:7570. doi: 10.1038/s41467-026-75789-w (PMC13415569; doi:10.1038/s41467-026-75789-w)
Supplement: Supplementary file 2 — Peer Review file [file 41467_2026_75789_MOESM2_ESM.pdf]

nature portfolio

Peer Review File

Machine Learning Reveals Biocontrol Agents Shaping Disease Outcome in Natural Arabidopsis Populations

Corresponding Author: Professor Eric Kemen

This file contains all reviewer reports in order by version, followed by all author rebuttals in order by version.

Version 0:

Reviewer comments:

Reviewer #1

(Remarks to the Author)
This manuscript investigates the relationship between infection status and phyllosphere microbiome dynamics in natural Arabidopsis populations over six generations, comparing plants infected by Albugo laibachii with uninfected controls. The authors use machine learning to identify disease-associated and health-associated microbial signatures, and test selected microbes for biocontrol activity against A. laibachii. The study culminates in validating the biocontrol capacity of Cystofilobasidium within a synthetic community derived from the Arabidopsis core microbiome.
Major Concerns
1. The manuscript lacks details on feature selection, hyperparameter tuning, and validation protocols. For example, how were OTUs prioritized for recursive feature elimination? Were nested cross-validation or independent test sets used to avoid overfitting?
2. Clarify why SVM, logistic regression (LR), and random forest (RF) were chosen. Include performance metrics (e.g., precision, recall, F1-score) beyond accuracy, which can be misleading in imbalanced datasets.
3 SPARCC is appropriate for compositional data, but the threshold for correlation significance (e.g., permutation-based p-values) is not specified. Provide adjacency matrices or code to ensure reproducibility.
4. The SynCom design (3 fungi, 12 bacteria) is not justified. Explain why these taxa were selected as the “core microbiome” and how their proportions reflect natural communities.
5. The claim that “site-dependent environmental factors rather than host genotypes” drive diversity loss (Lines 20–22) conflicts with prior studies showing host genetic influence on microbiomes. Reconcile this discrepancy using variance partitioning (e.g., PERMANOVA with genotype × site interactions).
6. The ML-identified “health-associated” microbes (e.g., Cystofilobasidium) are correlated with protection, but no mechanistic experiments (e.g., axenic plant inoculations, metabolite profiling) confirm their causal role. Include functional validation beyond co-inoculation assays.
7. The phylogenetic tree lacks bootstrap values, making evolutionary claims unreliable.
8. The SynCom experiments do not replicate natural microbial interactions. Discuss how simplified communities (15 strains) reflect the complexity of field microbiomes and whether SynCom efficacy scales to natural settings.
9. The study emphasizes Albugo but does not address how prior or co-occurring infections (common in nature) influence microbiome dynamics. Acknowledge this limitation.
10 Figure 2: box plots lack clarity. Use alpha diversity metrics (e.g., Shannon index) instead of “reduced microbial diversity” in text.
11. Figure 3: Network visualization is cluttered. Highlight keystone taxa/modules and simplify edge displays.
12. Multiple typos (e.g., “protec8on,” “pabern”) and inconsistent formatting (e.g., “Albugo laibachii” vs. “A. laibachii”).

Minor Concerns
1. Provide raw sequencing data (NCBI BioProject accession) and code for ML/network analyses (GitHub/Synapse).
2. Expand Supplementary Table S3 to include OTU sequences and alignment parameters.
3. Cite recent studies on ML in microbiome-based biocontrol.
4. Add a flowchart summarizing the experimental and computational workflow.
5. Clarify ML model validation and statistical thresholds for network edges.

6. Perform gnotobiotic experiments to test individual strains (e.g., Cystofilobasidium) in axenic plants.
7. Use metabolomics to identify antimicrobial compounds from “health-associated” microbes.

8. Discuss how environmental factors (e.g., soil pH, climate) interact with host genotype to shape microbiomes.
9. Address scalability of SynComs to agricultural systems.
10. Revise figures for clarity (e.g., label axes, add legends).
11. Proofread for typographical errors and formatting consistency.

(Remarks on code availability)

Reviewer #2

(Remarks to the Author)
The authors have employed a machine learning approach to identify microbiome signatures of healthy and diseased Arabidopsis plants. They test signatures of healthy plants for biocontrol activity and find that they can alleviate disease symptoms. In addition, they test a synthetic community based on the core microbiome and observe that adding an isolate from the genus Cystofilobasidium improves the disease outcome even further. Overall, the study is robust and based on a substantial dataset. However, the integration of the machine learning approach makes the impression that it was put on top of a well established knowledge base to extract information that would have very likely extractable with conventional approached. Therefore, my main suggestion would be to clearly show the value of the machine learning-based approach as compared to other approaches. It is clearly presented as a core and integral part of this study, but it is unclear if it is just a ‘fancy’ addon to bring something well established into new light or if it is of true values.

To address my main point, I would suggest to assess and describe the following based on the datasets that are already available:

-Does the machine learning approach result in the identification of different signatures than differential abundance analysis would have? If yes, to which extend does it so?

-Does the machine learning approach result in the identification of different signatures than conventional bioinformatic tools for biomarker discovery analysis such as LEfSe and other commonly used ones would? If yes, to which extend does it so?

-Qualitatively and quantitatively compare the machine learning approach to conventional approach that are used in microbiome research.

It would be crucial to clearly show that the machine learning-based approach is better than what is currently being used in this research field and substantiate this with experiment-based arguments.

It is well known that infected hosts have a different microbiome than healthy hosts after infection with various pathogens, which in most cases is also accompanied by reduced diversity. Are there any particular differences for the observations described here with Arabidopsis? It is not surprising that the microbiome of diseased plants had signatures that were stronger than the genotype as the pathogen itself likely substantially shapes the environment for microorganisms. In order to further expand on this, it would have been useful if the authors would have explored what exactly happens that leads to this microbiome shift. Is it increased leakage caused by the pathogen damaging leaf tissues? Is it the pathogens metabolites or degrading plant material that affects and shifts the microbiome under disease?

Minor comments:

Introduction: The breath of knowledge and research being conducted in the biocontrol sphere is not well shown. Reading the first paragraph makes the impression that it’s limited to a few observations and not a large and decades-old research field. Showing this would be useful for readers that are not familiar with the field.

Why was the analysis conducted on OTU level and not with a more precise ASV-based approach that would also allow better comparability with other studies?

The observations from the network analysis seem to be over-interpreted as they only rely on general observations such as connectivity of the nodes and other parameters that were not experimentally validated.

(Remarks on code availability)

Reviewer #3

(Remarks to the Author)
This manuscript: " Machine Learning Reveals Biocontrol Agents Shaping Disease Protection in Natural Arabidopsis Populations and Synthetic Communities" investigates the phyllosphere and endophytic microbiome composition associated with Albugo infection in Arabidopsis thaliana. The authors simultaneously characterized bacterial, fungal, and protistan communities, providing a comprehensive analysis of leaf-associated microbial dynamics under pathogen invasion. Several

key conclusions were drawn: 1） Leaf microbiome composition correlates more strongly with environmental factors than with Arabidopsis ecotypes.2） Protistan communities exhibited the most significant shifts during infection, with one protist strain reducing disease incidence by ~20%. 3） The proposed synthetic microbial community (SynCom) uniquely incorporates cross-kingdom diversity, distinguishing it from conventional designs. While the study demonstrates substantial efforts in both multi-omics analyses (16S/ITS/18S sequencing) and experimental validation (qPCR and infection assays), the overloaded presentation obscures critical aspects:
• The selection criteria for machine learning-identified biocontrol agents (e.g., Methylobacterium OTU3 and Cystofilobasidium OTU126) lack clear justification in SynCom design;
• The absence of cross-kingdom interaction analysis (e.g., bacterial-fungal-protozoan networks) represents a missed opportunity to elucidate the mechanisms underlying this innovative consortium design.
Major:
1 line 32:"This study reveals how microbial dynamics and functional connectivity support plant protection, ····" While the manuscript demonstrates comprehensive profiling of cross-kingdom microbiota (bacteria, fungi, and protists) in response to Albugo infection (Figs. 1-3), it lacks substantive analysis of inter-domain microbial interactions, Although those is very attractive.
2 line 50: "highlighting the need for reliable, standardized screening methods to enable the practical application of biocontrol agents····" The manuscript lacks a summary of the standardized methods for selecting combined bacterial communities.
3 line 64: "However, the application of machine learning to identify microbiome features linked to plant disease in natural settings remains underexplored····"The author lacks a description in the main text of the increased understanding of how machine learning identifies plant-associated microbes.

4 line 113： While PERMANOVA analysis revealed statistically significant effects of infection status on microbiome composition, the relatively low explanatory power (R^2=1.8-12.8%) may reflect the binary grouping approach (infected vs. uninfected). To better characterize these relationships, we recommend: Conducting stratified PERMANOVA analyses that account for: 1) Temporal variation (by sampling year),2)Spatial heterogeneity (by collection site).Then, calculating weighted average explanatory values across these stratified comparisons.

5: lines204-205: Why the modularity of the network may be related to infection status requires further explanation. And line 207:"communities to divide into distinct functional groups or ecological niche" further explanation is needed. For the modular analysis of microbial co-occurrence networks, need further explore the microbial composition of different modules and their functional similarities. This approach may better reveal key microbial groups related to plant health than simply calculating the modularity index. For more analyse network module analysis, we suggest implementing the ggClusterNet R package developed by Wen et al. (2022 iMeta).

6 I am very curious about how the strains selected by the author match the features microbes identified through amplicon microbiome analysis. To my knowledge, 16S rDNA is insufficient to support this matching.

Mini:
1 line 450: "The OTU tables of bacteria, fungi, and nonfungal eukaryotes were converted into relative" The microbial community composition may vary across kingdoms; for example, bacterial abundance is much higher than fungal abundance. Is it appropriate to standardize all data using relative abundance?
2 Fig3A : The colors of the lines representing positive and negative correlations in the figure are similar, making it difficult to read.
3 Fig2D: The stress value of the NDS analysis is not shown. Does it indicate a good fit?

(Remarks on code availability)

Version 1:

Reviewer comments:

Reviewer #1

(Remarks to the Author)
Major points
1. The title emphasizes the use of machine learning, but the abstract does not specify any particular algorithms, and the main text lacks a detailed description of the models, methods, and rationale for why this approach is more effective than conventional analyses. The authors should clearly describe each model, including parameters, training and validation strategies, and provide justification for why machine learning is appropriate and advantageous in addressing the research question.
2. Four machine learning methods are mentioned, but the rationale for selecting exactly four is unclear, and no discussion is provided regarding other approaches such as deep learning. The authors should explain the criteria for method selection and consider either justifying why deep learning was not applied or discussing its potential relevance.
3. Figure 1a does not convey meaningful information, and the description of the Arabidopsis population dataset is insufficient. The figure should be revised to present interpretable and relevant data, such as sample distributions or phenotype summaries, and the dataset should be described in detail, including population size, genotypes, growth

conditions, and preprocessing steps.
4. The machine learning pipeline shown in Figure 4a is incomplete and does not reflect a full analytical workflow. The figure and accompanying text should be expanded to include all steps, from data preprocessing and feature selection to model training, evaluation, and interpretation, ensuring the pipeline is complete and reproducible.
5. The code provided on the project website is unclear and appears to include only plotting scripts. The authors should provide complete, well-documented scripts covering data preprocessing, model training, evaluation, and visualization, along with a README file to guide reproduction of the results.
6. Genome analysis suggests potential terpene biosynthesis, but there is no direct evidence supporting antimicrobial activity. The authors should provide experimental or bioinformatic validation linking terpene pathways to antimicrobial compounds or explicitly present these results as preliminary and hypothesis-generating.

(Remarks on code availability)
The code provided on the website is not clear; it appears to include only some plotting scripts.

Reviewer #2

(Remarks to the Author)
The additional bioinformatic analyses are convicing and confirm the value of the ML approach. Overall, the study is robust and can serve as a blueprint for upcoming research aimed at implementing ML for the exploration of microbiomes.

Minor suggestions:
Figure 4 combines an overview of the analysis method and table elements. It might be better to split this into a clean schematic overview and include detailed parameters shown in panel "b" as a supplementary table.

Panel "a" in Figure 6 could be improved to become visually more appealing. Particularly the image sizes showing the plants could be increased. Letters indicating statistical significance in panel "g" are much smaller than those in the other panels of the same figure.

(Remarks on code availability)
The code was properly deposited and the additional information provided on gitlab facilitates reproducibility.

Reviewer #3

(Remarks to the Author)
The authors have carefully addressed the major and minor concerns raised during the first round of review. In particular, the revisions substantially clarify the rationale and implementation of the machine learning framework, improve transparency of model validation and feature selection, and better integrate the computational analyses with the experimental biocontrol assays. Overall, the revised manuscript is clearer, more balanced, and significantly strengthened compared to the previous version.

I have no remaining major concerns and support publication of the manuscript in its current form. I list below a few minor, optional points that the authors may consider addressing for further clarity, but none of these affect the main conclusions of the study.

Clarification of the added value of machine learning.
The revised text now more clearly positions the machine learning approach relative to conventional differential abundance and biomarker analyses. For maximal clarity to a broad readership, the authors may consider adding one or two concise summary sentences in the Discussion explicitly highlighting when and why the machine learning approach provides complementary or superior insights compared to traditional methods.

In summary, this is a well-executed and timely study that convincingly demonstrates how microbiome-informed machine learning can guide the identification of biocontrol agents in natural plant populations. I am pleased to recommend the manuscript for acceptance.

(Remarks on code availability)

Version 2:

Reviewer comments:

Reviewer #1

(Remarks to the Author)
No

(Remarks on code availability)

No

Reviewer #2

(Remarks to the Author)
I have only raised mionor points in the previous version, and I'm happy with how they were addressed.

(Remarks on code availability)
I didn't check this later on

Reviewer #3

(Remarks to the Author)
The authors have carefully addressed my previous concerns. The revised manuscript now provides a substantially clearer description of the machine-learning framework, including the algorithms used, model-selection rationale, validation strategies, hyperparameter optimization, and code availability. The revised figures and expanded Methods also improve the transparency and reproducibility of the computational analyses. I also appreciate the more cautious interpretation of the terpene-related genomic findings, which are now appropriately presented as preliminary and hypothesis-generating.

I have no remaining major concerns. I only suggest two minor points for clarity. First, the authors should explicitly state whether RFECV-based feature selection was performed strictly within the training folds during cross-validation, to avoid any potential information leakage. Second, unless additional metabolite-level or functional evidence is provided, the wording around terpene-related antimicrobial activity should remain cautious; for example, “potential candidates” may be preferable to “strong candidates”.

Overall, the revision has substantially strengthened the manuscript, and I support publication after these minor clarifications.

(Remarks on code availability)

Open Access This Peer Review File is licensed under a Creative Commons Attribution 4.0 International License, which permits use, sharing, adaptation, distribution and reproduction in any medium or format, as long as you give appropriate credit to the original author(s) and the source, provide a link to the Creative Commons license, and indicate if changes were made.
In cases where reviewers are anonymous, credit should be given to 'Anonymous Referee' and the source.
The images or other third party material in this Peer Review File are included in the article’s Creative Commons license, unless indicated otherwise in a credit line to the material. If material is not included in the article’s Creative Commons license and your intended use is not permitted by statutory regulation or exceeds the permitted use, you will need to obtain permission directly from the copyright holder.
To view a copy of this license, visit https://creativecommons.org/licenses/by/4.0/

REVIEWER COMMENTS

Reviewer #1 (Remarks to the Author):

This manuscript investigates the relationship between infection status and phyllosphere microbiome dynamics in natural Arabidopsis populations over six generations, comparing plants infected by Albugo laibachii with uninfected controls. The authors use machine learning to identify disease-associated and health-associated microbial signatures, and test selected microbes for biocontrol activity against A. laibachii. The study culminates in validating the biocontrol capacity of Cystofilobasidium within a synthetic community derived from the Arabidopsis core microbiome.

Major Concerns

1. The manuscript lacks details on feature selection, hyperparameter tuning, and validation protocols. For example, how were OTUs prioritized for recursive feature elimination? Were nested cross-validation or independent test sets used to avoid overfitting?

>> We thank the reviewer for this great suggestion. We have now optimized and included all details of feature selection, hyperparameter tuning, and validation protocols in the main text (Lines: 257-272) and Methods section (Lines: 566-603). We also updated the schematic overview of the analysis steps in Fig. 4a.

Specifically:

1) **We optimized the hyperparameter tuning using GridSearchCV with 5-fold cross-validation which improved robustness of results and identified features significantly (Fig. 4&5).**
2) **We validated the model performance with three complementary strategies to ensure robust assessment and minimize overfitting: a 70:30 train–test split, where models were trained on 70% of the data and tested on 30%; 5-fold cross-validation with partial nesting, where Recursive Feature Elimination with Cross-Validation (RFECV) selected optimal features within folds; and leave-one-out (LOO) cross-validation, where each sample was held out in turn and the model trained on the remaining samples.**
3) **We clarified that OTUs were prioritized for Recursive Feature Elimination based on their contribution to cross-validation accuracy and feature importance metrics derived from the classifiers.**

After the benchmarking of different training and analysis pipelines with more comprehensive metrics, we found all different machine learning pipelines could achieve a decent success rate in separating infected and uninfected samples, consistent with the previous version. However, the reviewer's suggestion revealed that the SVM algorithm with LOO cross-validation resulted in the highest level of overall accuracy, which is better than what we found from the previous version (LR with 70:30 split). We therefore updated the corresponding result section (Lines: 282-284) and sincerely thank the reviewer again for this excellent suggestion.

2. Clarify why SVM, logistic regression (LR), and random forest (RF) were chosen. Include performance metrics (e.g., precision, recall, F1-score) beyond accuracy, which can be misleading in imbalanced datasets.

>> We thank the reviewer for this helpful comment, which significantly enriched the depth and reliability of our machine learning analysis.

We have now clarified the rationale of selecting these models at Lines: 254–257:

These models were selected based on their ability to capture both linear (SVM and LR) and non-linear (RF and MLP) relationships, and prior success in microbiome-based classification tasks [Marcos-Zambrano et al, 2021].

As suggested by the reviewer, we have included different performance metrics such as precision, recall, F1-score, and AUC in addition to accuracy for better assessment of different machine learning models. We have updated Fig. 4b and added the results from additional metrics in Fig. 4c. We also adjusted the corresponding texts from Lines: 273-286.

3 SPARCC is appropriate for compositional data, but the threshold for correlation significance (e.g., permutation-based p-values) is not specified. Provide adjacency matrices or code to ensure reproducibility.

>> We thank the reviewer for this suggestion. All scripts and outputs, including adjacency matrices containing information on all possible edges, have now been added to the GitLab repository to ensure reproducibility (median_correlation.tsv and pvalues.tsv for infected and uninfected networks). We have also included the correlation significance thresholds used for network construction in this study and clarified this in the legend of Fig. 3 and method section ($p \leq 0.01$ and $|r| \geq 2$, Supplementary Table 5) (Lines: 631–634).

4. The SynCom design (3 fungi, 12 bacteria) is not justified. Explain why these taxa were selected as the "core microbiome" and how their proportions reflect natural communities.

>> We thank the reviewers for their constructive comments regarding the design and ecological validity of our SynCom. We have revised the manuscript to better justify our SynCom design and acknowledge the limitation:

1. We performed additional analysis (Supplementary Fig. 7b) and provided more details on the selection of SynCom members in the result section:

Lines: 413-418: We further evaluated the protective effects of *Cystofilobasidium* in a community context using a simplified synthetic microbial community (SynCom) consisting of 15 members (12 bacteria and 3 fungi). This SynCom was designed based on the Arabidopsis core microbiome, defined by the presence of OTUs in more than 95–98% of samples collected over three years of garden experiments (Supplementary Fig. 7a). It was previously shown to be stably reproduced both in vitro and in planta, displaying tolerance to metabolite perturbations. In our current dataset, most OTUs corresponding to SynCom members were identified in the majority of samples (Supplementary Fig. 7b). Given its in planta stability and ecological relevance to our dataset, we employed this SynCom to test the effect of *Cystofilobasidium* in a community context.

The corresponding method section is also updated (Lines: 663-666)

2. Regarding the community proportions, the composition (12 bacteria and 3 fungi) was specifically selected to reflect the natural dominance of bacterial diversity and abundance over fungi in the *Arabidopsis* phyllosphere. We have clarified this in the Methods section:

Lines: 663-666: SynCom members consisted of 12 bacterial strains and three yeasts were selected to reflect the natural dominance of bacterial diversity and abundance over fungi in the Arabidopsis phyllosphere, ensuring the SynCom mirrors the kingdom-level balance observed in the field.

3. We revised the discussion to have a more balanced tone about the design and use of SynCom, which they do not fully capture the diversity in natural communities, but is useful in representing the 'core' microbiome:

Lines: 508-515: Synthetic communities provide a powerful tool to bridge the gap between in vitro assays and complex field phenotypes (Paredes et al., 2018). We acknowledge that our 15-member SynCom is a reductionist model that does not fully capture the extensive taxonomic variation and complexity of the natural phyllosphere microbiome. However, by constructing the community based on the persistent 'core' microbiome, we targeted taxa with high ecological stability—a strategy increasingly recognized for developing robust biocontrol solutions.

5. The claim that "site-dependent environmental factors rather than host genotypes" drive diversity loss (Lines 20–22) conflicts with prior studies showing host genetic influence on microbiomes. Reconcile this discrepancy using variance partitioning (e.g., PERMANOVA with genotype × site interactions).

>> We thank the reviewer for raising this point, which we also found somewhat surprising. As suggested, we included variance partitioning results using the model ~ Site * Genotype. These results indicate that both sampling site and host genotype contribute similarly to shaping microbial community composition, with host genotype explaining 6–10.9% of the variance and sampling site accounting for up to 11.7%; the site × genotype interaction was not significant (Supplementary Table 2; Lines: 170-192).

We think this observation is reasonable and potentially interesting. Host genetic effects on microbiome composition have been reported in various contexts, particularly in crop systems (Rodríguez et al., 2025). In contrast, our data come from the phyllosphere microbiome of *Arabidopsis* sampled under natural conditions over six years, during which the climate varied substantially between spring and fall. Moreover, the phyllosphere of the annual plant *A. thaliana* is a transient ecological niche (Vorholt, 2012). Therefore, we believe it is important to consider the temporal scale when assessing host genetic effects on the microbiome. Incorporating time explicitly—especially in agricultural settings—may be essential for accurately evaluating how strongly host genotype shapes microbiome composition in a fluctuating environment.

Since the primary goal of our study is to identify biocontrol agents, we only present these observations in the Results section, while maintaining focus on biocontrol identification. However, we added our thoughts in the Discussion with a more balanced perspective, emphasizing that both genotype and site influence microbiome composition (Lines: 461-470).

6. The ML-identified "health-associated" microbes (e.g., Cystofilobasidium) are correlated with protection, but no mechanistic experiments (e.g., axenic plant inoculations, metabolite profiling) confirm their causal role. Include functional validation beyond co-inoculation assays.

>> We thank the reviewer for this suggestion. We have now included new experiments to explore the mechanistic insight on how *Cystofilobasidium* reduces *Albugo* infection.

Firstly, we performed a gnotobiotic infection assay to test if *Cystofilobasidium* inhibits Albugo colonization via priming plant defence. The results indicated that *Cystofilobasidium* acts more like a commensal yeast and does not induce plant defence response. In this case, we think that it inhibits *Albugo* colonization via microbe-microbe interactions, which could be either direct or indirect inhibition.

To investigate the potential mechanism of microbe-microbe interactions, we next performed whole genome sequencing of three different strains of *Cystofilobasidium macerans* (within OTU126), plus two close related species *C. capitatum* and a *Tausonia* pullulans strain, all of which are isolated from the sampling sites around Tuebingen. We found that genomes of these strains contain several terpenes related metabolic pathways, indicating its potential role in inhibiting *Albugo* colonization.

We thus revised the result section and added the new data in Fig. 6d-f: Lines: 413-425:

We are currently running another major project trying to dissect the molecular mechanisms of how *Cystofilobasidium* inhibits *Albugo* infection. We found that Cystofilobasidium can cooperate with some *Pseudomonas* strains and boost their capacity of producing antimicrobial compounds. We have performed the metabolomics experiment in this project, but since the mechanism is rather complicated and not directly related with the main message of this manuscript, we sought to not include it in this revision. However, if the reviewer thinks that this is necessary, we are happy to add the *in vitro* metabolomics result.

7. The phylogenetic tree lacks bootstrap values, making evolutionary claims unreliable.

>> We thank the reviewer for pointing this out. We have now added bootstrap values to the phylogenetic tree in Supplementary Fig. 7a to provide statistical support for each branch.

8. The SynCom experiments do not replicate natural microbial interactions. Discuss how simplified communities (15 strains) reflect the complexity of field microbiomes and whether SynCom efficacy scales to natural settings.

>> We agree with the reviewer that a 15-member community is a reductionist model that cannot fully capture the complexity of the natural phyllosphere microbiome. We have expanded our Discussion to openly acknowledge this limitation as shown in the response to the major comment #4:

Lines: 509-511: We acknowledge that our 15-member SynCom is a reductionist model that does not fully capture the extensive taxonomic variation and complexity of the natural phyllosphere microbiome.

Nevertheless, we consider this reductionism essential to dissect causal relationships that are often obscured by environmental noise in field settings. By using a defined "core" community, we model the fundamental cross-kingdom interactions that persist across environments. The fact that *Cystofilobasidium* significantly enhanced protection *within* this community context (Fig. 6g) provides robust evidence that its biocontrol activity is not an artifact of isolation but is synergistic with resident "core" microbes. This step—validating efficacy in a community context—is a critical bridge between *in vitro* assays and complex natural settings. Therefore, we also added the justification in the same paragraph of discussion to ensure a balanced tone:

Lines: 511-517: However, by constructing the community based on the persistent 'core' microbiome, we targeted taxa with high ecological stability—a strategy increasingly recognized for developing robust biocontrol solutions [43, 44]. Consistent with the view that core communities can act as a barrier against pathogen invasion [43], the SynCom alone yielded a moderate reduction in Albugo infection. The addition of Cystofilobasidium further enhanced this protective effect, demonstrating its synergistic contribution to community-level defense.

9. The study emphasizes Albugo but does not address how prior or co-occurring infections (common in nature) influence microbiome dynamics. Acknowledge this limitation.

>> We agree with the reviewer that natural plant populations frequently experience co-infections, and the interaction history between multiple pathogens can significantly shape microbiome assembly. While our study focused on *Albugo* due to its dominance and distinct symptomatology in this population, we acknowledge that we did not explicitly quantify prior or co-occurring infections by other pathogens (e.g., viruses or asymptomatic bacterial pathogens). These unmeasured biotic interactions likely contribute to the unexplained variance observed in our diversity analyses. We have added a statement in the Discussion section to explicitly acknowledge this limitation.

Lines: 470-475: Beyond these drivers, natural populations frequently experience varying histories of prior or co-occurring infections; such unmeasured biotic interactions likely contribute to the remaining unexplained variability. Collectively, these findings highlight the context-dependent nature of plant microbiome assembly, shaped by dynamic interactions between environmental conditions, host genotype, and diverse biotic interactions.

10. Figure 2: box plots lack clarity. Use alpha diversity metrics (e.g., Shannon index) instead of "reduced microbial diversity" in text.

>> We thank the reviewer for this suggestion, which further clarified our results. We have now updated Fig. 2 and the corresponding text to report the Shannon diversity index instead of the general term "reduced microbial diversity".

11. Figure 3: Network visualization is cluttered. Highlight keystone taxa/modules and simplify edge displays.

>> We thank the reviewer for this nice suggestion. We improved the network visualization in Fig. 3a&b and Supplementary Fig. 3 by (i) highlighting the microbes identified through our machine learning analysis; (ii) simplifying the network by applying stricter thresholds

(|r| ≥ 0.2, p ≤ 0.01) to retain most significant correlations and reduce edge density; and (iii) improving the display of the network's modular structure, while avoiding cutoffs that would make the network too sparse to detect modules. These adjustments reduced clutter and made keystone taxa and modules more visible.

12. Multiple typos (e.g., "protec8on," "pabern") and inconsistent formatting (e.g., "Albugo laibachii" vs. "A. laibachii").

>> We sincerely apologize for these mistakes. All typographical errors and formatting inconsistencies have been carefully corrected throughout the revised manuscript.

Minor Concerns

1. Provide raw sequencing data (NCBI BioProject accession) and code for ML/network analyses (GitHub/Synapse).

>> We have now updated the 'Availability of data and material' which includes path of GitLab for all the scripts and files and BioProject ID for the sequences: Lines: 806-812,

2. Expand Supplementary Table S3 to include OTU sequences and alignment parameters.

>> The former Supplementary Table 3 has now been relabelled as Supplementary Table 4 and updated to include OTU sequences together with detailed BLAST search parameters (program type, database, and E-value threshold) and corresponding alignment results to clarify taxonomic assignment procedures.

3. Cite recent studies on ML in microbiome-based biocontrol.

>> We thank the reviewer for this comment. We have added studies in the Introduction highlighting the application of machine learning to microbiome-based plant health and biocontrol (Lines: 67–77). These include examples of ML predicting outcomes of various plant pathogens, linking microbiome composition to crop productivity, identifying bacterial strains that suppress infections, and forecasting plant vigor or soil health based on microbial profiles.

4. Add a flowchart summarizing the experimental and computational workflow.

We thank the reviewer for this excellent suggestion. We have now generated a schematic overview of the whole computational and experimental workflow in Supplementary Fig. 8.

5. Clarify ML model validation and statistical thresholds for network edges.

>> We thank the reviewer for this suggestion. All details regarding parameter tuning for machine learning models (Lines: 566-602) and the criteria used to select significant edges in the network (Lines: 633-637) statistics have been added to the Methods section and text.

6. Perform gnotobiotic experiments to test individual strains (e.g., Cystofilobasidium) in axenic plants.

>> We thank the reviewer for this suggestion. We have added this experiment as described above in response to the major comment #6

7. Use metabolomics to identify antimicrobial compounds from "health-associated" microbes.

We thank the reviewer for the good suggestion. As mentioned above, we think the first step forward is to identify potential direction of how does *Cystofilobasidium* reduce *Albugo* colonization, since it can be through not only metabolites, but also other mechanism such as biofilm formation, direction inhibition, or protein mediated inhibition. Therefore, instead of performing metabolomics, we sequenced the genome of different *Cystofilobasidium* strains and tried to first identify the potential mechanism of the biocontrol activities.

We are currently running a follow-up project to dissect the molecular mechanisms of the microbial interaction centred by *Cystofilobasidium*, which we think is a separate study and deserves a different paper. Therefore, we decided to only report the genome results in this work, highlighting the mechanistic insights on the biocontrol function of *Cystofilobasidium.*

8. Discuss how environmental factors (e.g., soil pH, climate) interact with host genotype to shape microbiomes.

>> We thank the reviewer for this suggestion. We have now expanded the discussion to integrate recent studies (Lines: 464-470) showing that soil and climate are dominant drivers of microbial variation for the microbiome *Arabidopsis* and several crop species, while host genotype can also modulate community structure, sometimes in interaction with environmental conditions. This addition highlights the complex interplay between environment and genetics in structuring both root and leaf microbiomes.

9. Address scalability of SynComs to agricultural systems.

>> We agree that scalability is a key consideration. We believe that targeting the "core microbiome" supports scalability by prioritizing taxa with proven persistence across varying environments (Toju et al., 2018). We have added a concluding sentence to the Discussion to explicitly link our findings to this ecological framework:

Lines 517-520: "Ultimately, this focus on the core microbiome addresses a major challenge in scalability: by selecting ubiquitous and adaptable taxa, we align with ecological frameworks for designing resilient microbiomes for sustainable agroecosystems [45]."

10. Revise figures for clarity (e.g., label axes, add legends).

>> We thank the reviewer for raising this point, and sincerely apologize for our mistakes. All figures have been revised for improved clarity. Axis labels, legends, and consistent formatting have been added or adjusted as needed. We will also work with the professional team from the journal to cross-check our revision.

11. Proofread for typographical errors and formatting consistency.

>> We sincerely apologize for these mistakes. The entire manuscript has been thoroughly proofread to correct typographical errors and ensure consistent formatting throughout.

Reviewer #2 (Remarks to the Author):

The authors have employed a machine learning approach to identify microbiome signatures of healthy and diseased Arabidopsis plants. They test signatures of healthy plants for biocontrol activity and find that they can alleviate disease symptoms. In addition, they test a synthetic community based on the core microbiome and observe that adding an isolate from the genus Cystofilobasidium improves the disease outcome even further. Overall, the study is robust and based on a substantial dataset. However, the integration of the machine learning approach makes the impression that it was put on top of a well established knowledge base to extract information that would have very likely extractable with conventional approached. Therefore, my main suggestion would be to clearly show the value of the machine learning-based approach as compared to other approaches. It is clearly presented as a core and integral part of this study, but it is unclear if it is just a 'fancy' addon to bring something well established into new light or if it is of true values.

To address my main point, I would suggest to assess and describe the following based on the datasets that are already available:

-Does the machine learning approach result in the identification of different signatures than differential abundance analysis would have? If yes, to which extend does it so?

-Does the machine learning approach result in the identification of different signatures than conventional bioinformatic tools for biomarker discovery analysis such as LEfSe and other commonly used ones would? If yes, to which extend does it so?

-Qualitatively and quantitatively compare the machine learning approach to conventional approach that are used in microbiome research.

It would be crucial to clearly show that the machine learning-based approach is better than what is currently being used in this research field and substantiate this with experiment-based arguments.

>> We thank the reviewer for this valuable suggestion. We have used current state-of-art tools, including LEfSe and an independent Wilcoxon rank-sum test to identify the biomarker differentiating the infected and uninfected samples, and compared it with our machine learning based methods (Results, Lines: 311–323; Supplementary Fig. 4d; Supplementary Table 4; Methods, Lines: 604-619). We decided to not perform the differential abundance approaches since it has been previously suggested that they provide inconsistent results across various datasets (Nearing et al., 2022).

In the LEfSe analysis, the statistical step initially identified 235 taxa with significant differences between groups; however, none exceeded the default LDA threshold of > 2.0, indicating that although minor abundance differences exist, no individual taxon showed a strong discriminative effect (Supplementary Table 4). The external Wilcoxon test produced

a similar list of significant taxa ($p < 0.05$). These strategies are based on effect from individual taxa, which is often not obvious given a community context.

In contrast, our machine-learning (ML) approach identified 56 predictive microbial features, of which five were consistently shared across all ML models (Supplementary Fig. 4c) and four overlapped with Wilcoxon-identified taxa (Supplementary Fig. 4d). This is a shorter list of features compared to the number identified by the conventional approaches, exhibiting a quantitative advantage during the screening.

Qualitatively, ML-based models capture patterns that arise from changes in multiple organisms simultaneously. This enables the detection of community-level interactions and non-linear relationships among taxa that together best discriminate between sample classes, resulting in feature sets that are likely more biologically meaningful. Notably, all strains selected from the ML-derived signature and tested experimentally were able to reduce *Albugo* infection, further supporting the functional relevance of the ML-identified patterns. In addition, the machine-learning approach learns patterns from a training dataset and, once validated, accurately predicts the status of new samples. Traditional univariate or rule-based methods do not offer this predictive capability.

We also added the above points in the discussion at Lines: 491-498.

It is well known that infected hosts have a different microbiome than healthy hosts after infection with various pathogens, which in most cases is also accompanied by reduced diversity. Are there any particular differences for the observations described here with Arabidopsis? It is not surprising that the microbiome of diseased plants had signatures that were stronger than the genotype as the pathogen itself likely substantially shapes the environment for microorganisms. In order to further expand on this, it would have been useful if the authors would have explored what exactly happens that leads to this microbiome shift. Is it increased leakage caused by the pathogen damaging leaf tissues? Is it the pathogens metabolites or degrading plant material that affects and shifts the microbiome under disease?

>> We thank the reviewer for raising this important point.

We attempted to obtain the big picture and explore the cause of the microbiome shift from three aspects:

1. To further validate that infection status is the key factor responsible for the microbiome shift, we performed PERMANOVA analysis within each 'sampling year' (Line: 115-118), 'host genotype' (Lines: 170-172) and 'sampling site' (Lines: 183-192). We found that infection status still explains a decent percentage of variation within each factor, which is higher or comparable with the percentage of variation explained by each factor themselves. This result further supports that *Albugo* infection is the key driver of the microbiome shift.

2. We investigated whether the microbiome shift was purely pathogen-driven or involved a plant-mediated "cry-for-help" recruitment. Therefore, we explored the mode-of-act of the identified biocontrol candidate *Cystofilobasidium*. We found that this strain does not induce plant defence response. We further sequenced the genome of several *Cystofilobasidium* strains and identified gene clusters responsible for terpene synthesis, suggesting that *Cystofilobasidium* suppresses *Albugo* colonization through direct microbe-microbe competition rather than via host immunity (Lines: 397-412).

3. From the pathogen side, previous studies suggest that *Albugo* likely mediates these microbiome shifts through partial suppression of host immunity[41], reprogramming of the leaf metabolome[42], and the secretion of antimicrobial compounds that selectively inhibit competitors[43]. Unraveling precisely how pathogens actively engineer the leaf micro-environment to facilitate colonization represents a critical frontier in plant microbiome research. We have added this in the discussion Lines: 484-488.

Minor comments:

Introduction: The breath of knowledge and research being conducted in the biocontrol sphere is not well shown. Reading the first paragraph makes the impression that it's limited to a few observations and not a large and decades-old research field. Showing this would be useful for readers that are not familiar with the field.

>> We have revised the first paragraph to explicitly state that this field spans five decades of research. We have also expanded the description of known mechanisms of action (competition, antimicrobial production, and host defense induction) to provide a broader context for readers (Lines: 45-51).

Why was the analysis conducted on OTU level and not with a more precise ASV-based approach that would also allow better comparability with other studies?

>> We thank the reviewer for raising this point. Although ASV-based approaches offer higher taxonomic resolution, minimize sequencing errors, and can detect rare or novel taxa—particularly in bacterial datasets—our analysis followed a classical mothur-based workflow in which high-quality unique sequences are clustered into OTUs at 97% similarity. This approach is more robust for eukaryotic microbes, which often contain multiple divergent marker-gene copies and exhibit high sequence variability, and our dataset included several groups of eukaryotes. Therefore, we chose OTU clustering to balance resolution and comparability across all samples. Moreover, OTU-level resolution provides a better framework for network analysis across diverse microbial communities. Using ASVs directly would artificially split signals belonging to the same genus or species, potentially obscuring ecologically meaningful interactions. From a translational perspective—particularly for identifying potential biocontrol candidates—OTU or genus-level resolution is sufficient to narrow down candidate taxa, whereas requiring ASV- or strain-level precision at this stage could limit reproducibility and reduce the likelihood of matching isolates from existing culture collections. We have now clarified this rationale in the revised Methods section.

The observations from the network analysis seem to be over-interpreted as they only rely on general observations such as connectivity of the nodes and other parameters that were not experimentally validated.

>> We agree with the reviewer that network analyses reflect correlations rather than direct causal relationships. We have clarified in the Discussion (Lines: 479-486) that the observed patterns, such as differences in connectivity and modularity, highlight potential microbial associations and community restructuring under infection, but do not demonstrate direct

causation. These shifts may result from *Albugo*-mediated effects, including partial suppression of host immunity, alterations of the leaf metabolome (e.g., changes in tryptophan-derived antimicrobial metabolites), and secretion of antimicrobial compounds into the apoplast, which together could reshape the microbial environment.

Reviewer #3 (Remarks to the Author):

This manuscript: " Machine Learning Reveals Biocontrol Agents Shaping Disease Protection in Natural Arabidopsis Populations and Synthetic Communities" investigates the phyllosphere and endophytic microbiome composition associated with Albugo infection in Arabidopsis thaliana. The authors simultaneously characterized bacterial, fungal, and protistan communities, providing a comprehensive analysis of leaf-associated microbial dynamics under pathogen invasion. Several key conclusions were drawn: 1) Leaf microbiome composition correlates more strongly with environmental factors than with Arabidopsis ecotypes. 2) Protistan communi-ties exhibited the most significant shifts during infection, with one protist strain reducing disease incidence by ~20%. 3) The proposed synthetic microbial community (SynCom) uniquely incorporates cross-kingdom diversity, distinguishing it from conventional designs. While the study demonstrates substantial efforts in both multi-omics analyses (16S/ITS/18S sequencing) and experimental validation (qPCR and infection assays), the overloaded presentation obscures critical aspects:

• The selection criteria for machine learning-identified biocontrol agents (e.g., Methylobacterium OTU3 and Cystofilobasidium OTU126) lack clear justification in SynCom design;

>> We appreciate the reviewer's comment regarding the justification of our strain selection. Our selection strategy for the Synthetic Community (SynCom) and individual testing was driven by a combination of computational predictions and practical availability.

We have revised the Results section to clarify the selection criteria of candidate OTUs for experiments:

Lines: 335-339: The 56 key OTUs comprise 24 fungi, 20 bacteria, and 12 NFEuk. To experimentally validate their effects on *Albugo* infection, seven representative taxa were selected based on: (i) identification by at least two machine learning models, (ii) broad taxonomic diversity across kingdoms, and (iii) availability of isolates in culture collections.

Furthermore, to provide better context for how these agents were tested, we added an analysis in the Results (Lines: 413–418) justifying the ecological relevance of the background SynCom (based on the *Arabidopsis* core microbiome). We also expanded the Discussion (Lines: 508–518) to critically review the potential and limitations of using reductionist SynComs in microbiome studies.

• The absence of cross-kingdom interaction analysis (e.g., bacterial-fungal-protozoan networks) represents a missed opportunity to elucidate the mechanisms underlying this innovative consortium design.

>> We thank the reviewer for this excellent suggestion. We have now conducted new analyses of intra- and inter-kingdom correlations (Lines: 226–236; Supplementary Fig. 3) which showed increased cross-kingdom antagonism under *Albugo* infection. Full networks, including bacteria, fungi, and other eukaryotes, are available as Cytoscape files on GitHub for further exploration and validation by other researchers.

Major:

1 line 32:"This study reveals how microbial dynamics and functional connectivity support plant protection, ····" While the manuscript demonstrates comprehensive profiling of cross-kingdom microbiota (bacteria, fungi, and protists) in response to Albugo infection (Figs. 1-3), it lacks substantive analysis of inter-domain microbial interactions, Although those is very attractive.

>> We thank the reviewer for this insightful comment. We have now updated our network analysis based on different comments in order to improve its clarity and comprehensiveness:

1. We quantified both within- and between-domain (bacteria, fungi, Nonfungal eukaryotes) correlations in uninfected and infected networks (Lines: 226–236; Supplementary Fig. 3). Infection increased the total number of edges but also elevated negative correlations across kingdoms, indicating enhanced cross-kingdom antagonism and niche partitioning. These results show that infection influenced the microbial community into a more modular structure with coordinated interactions and changes across microbial kingdoms.

2. We improved the network visualization in Fig. 3a&b and Supplementary Fig. 3 by (i) highlighting the microbes identified through our machine learning analysis; (ii) simplifying the network by applying stricter thresholds ($|r| \geq 0.2$, $p < 0.01$) to retain most significant correlations and reduce edge density; and (iii) emphasizing modular structure. These adjustments reduced clutter and made keystone taxa and modules more visible.

2 line 50: "highlighting the need for reliable, standardized screening methods to enable the practical application of biocontrol agents····" The manuscript lacks a summary of the standardized methods for selecting combined bacterial communities.

>> We thank the reviewer for this suggestion. In response, we have added a flow chart (Supplementary Fig. 8) that summarizes the full computational and experimental workflow. The figure illustrates how candidate microbes were prioritized by integrating multiple machine-learning and statistical approaches, how putative interactions were assessed using network analysis, and how these candidates were subsequently validated experimentally—both as individual isolates and within synthetic communities.

3 line 64: "However, the application of machine learning to identify microbiome features linked to plant disease in natural settings remains underexplored····"The author lacks a description in the main text of the increased understanding of how machine learning identifies plant-associated microbes.

>> We have revised the third paragraph (Lines: 67-81) of the Introduction to more clearly describe how machine learning contributes to identifying plant-associated microbes. Specifically, we removed the statement that this area is "underexplored" and instead explained how machine-learning algorithms analyze high-dimensional microbiome data to capture complex, non-linear relationships that are difficult to resolve with traditional statistical approaches. We now emphasize that this enables the identification of predictive microbial features and signatures linked to plant health and disease outcomes, supported by multiple published examples.

4 line 113： While PERMANOVA analysis revealed statistically significant effects of infection status on microbiome composition, the relatively low explanatory power (R^2=1.8-12.8%) may reflect the binary grouping approach (infected vs. uninfected). To better characterize these relationships, we recommend: Conducting stratified PERMANOVA analyses that account for: 1) Temporal variation (by sampling year), 2) Spatial heterogeneity (by collection site). Then, calculating weighted average explanatory values across these stratified comparisons.

>> We thank the reviewer for this valuable suggestion. We have performed all suggested PERMANOVA analyses with updated Supplementary Table 2, and the corresponding text (Lines: 115-118,170-175,184-191) to show the stratified effects of infection in different years and sampling sites and weighted average explanatory values were calculated across these stratified comparisons. The results show slightly higher explanatory values compared to the previous analysis; for example, infection explained 4.7–15% of the variation when stratified by year.

5: lines204-205: Why the modularity of the network may be related to infection status requires further explanation. And line 207:"communities to divide into distinct functional groups or ecological niche" further explanation is needed. For the modular analysis of microbial co-occurrence networks, need further explore the microbial composition of different modules and their functional similarities. This approach may better reveal key microbial groups related to plant health than simply calculating the modularity index. For more analyse network module analysis, we suggest implementing the ggClusterNet R package developed by Wen et al. (2022 iMeta).

>> We thank the reviewer for this inspiring suggestion. We have now cited *Wen et al. (2022, iMeta)* as inspiration for improving network visualization and interpretation. As described in response to the major concern #1 above, we refined our co-occurrence network analysis and updated Fig. 3 to better illustrate the distribution of key taxa across modules. In addition, we performed complementary within- and between-kingdom network analyses (new Supplementary Fig. 3), which revealed predominantly positive associations within kingdoms and mainly negative associations between kingdoms, particularly in infected networks. These results provide a clearer ecological explanation of how infection status influences microbial modularity and interkingdom interactions (Lines: 226-236).

6 I am very curious about how the strains selected by the author match the features microbes identified through amplicon microbiome analysis. To my knowledge, 16S rDNA is insufficient to support this matching.

>> We thank the reviewer for raising this critical point, and we fully acknowledge that 16S/ITS amplicon sequences (short reads) cannot provide absolute strain-level resolution.

We selected isolates for validation by aligning the full-length 16S rRNA (for bacteria) or ITS (for fungi) sequences of our cultured isolates against the representative short-read sequences of the key OTUs identified by the machine learning models. We selected isolates with the highest sequence identity to the ML-identified features as the best available proxies.

We have revised the Results section to clarify our selection method and explicitly acknowledge this limitation:

Line 339-342: To link these isolates to the Machine Learning predicted OTUs, the full-length 16S rRNA or ITS sequences of our cultured isolates were aligned against the representative sequences of the key OTUs using BLASTn, and isolates with the highest sequence identity (closest match) were selected.

Lines: 346-348: Given the known phenotypic variability between strains of the same microbial species, we acknowledge that marker gene sequences alone do not fully inform on the functional potential of the selected strains (Karasov et al., 2018).

We also emphasized the selection process again in the corresponding Method section (Lines: 649-655).

Mini:

1 line 450: "The OTU tables of bacteria, fungi, and nonfungal eukaryotes were converted into relative" The microbial community composition may vary across kingdoms; for example, bacterial abundance is much higher than fungal abundance. Is it appropriate to standardize all data using relative abundance?

>> We thank the reviewer for highlighting this critical methodological detail. We understand the concern that normalizing kingdoms separately might artificially inflate the weight of lower-abundance groups (like fungi) relative to high-abundance groups (like bacteria). To clarify, we calculated the relative abundance independently within each kingdom (Bacteria, Fungi, and Other eukaryotes) before merging them into a single table. By normalizing each dataset independently (so that each kingdom sums to 1 per sample), we ensured that the variation in sequencing depth or total abundance between kingdoms did not bias the analysis. This approach preserves the data structure of lower-abundance kingdoms (like fungi) and ensures they contribute equally to the feature space before the Machine Learning classifiers are applied.

From a Machine Learning perspective, our goal is to identify biomarkers that predict infection, regardless of their total biomass. A low-abundance fungal pathogen or antagonist can be just as biologically potent as a high-abundance commensal bacterium (the "Keystone Species" concept Banerjee et al., 2018). If we did not normalize kingdoms separately, bacterial features would numerically dominate the dataset, potentially causing the model to overlook highly predictive fungal signals. By scaling each kingdom to the same range, we

ensure that the classifier evaluates features based on their predictive power (their association with infection) rather than their raw abundance.

We have clarified this step in the Methods section:

Lines: 556-559: "The OTU tables of bacteria, fungi, and non-fungal eukaryotes were independently converted to relative abundance within each kingdom and subsequently merged into a single composite table. This ensured that cross-kingdom differences in sequencing depth and amplification efficiency did not bias the feature selection."

2 Fig3A : The colors of the lines representing positive and negative correlations in the figure are similar, making it difficult to read.

>> We thank the reviewer for this good suggestion and apologize for the confusing illustration. We have adjusted the edge colours in Fig. 3a and retain only significant correlations to improve readability.

3 Fig2D: The stress value of the NDS analysis is not shown. Does it indicate a good fit?

>> The NMDS stress values were 0.21–0.28, indicating an acceptable fit, and Procrustes analysis confirmed consistency among runs. We have added these values to all related figures including Fig. 1c and Fig. 2b&d.

REVIEWER COMMENTS

Reviewer #1 (Remarks to the Author):

Major points

1. The title emphasizes the use of machine learning, but the abstract does not specify any particular algorithms, and the main text lacks a detailed description of the models, methods, and rationale for why this approach is more effective than conventional analyses. The authors should clearly describe each model, including parameters, training and validation strategies, and provide justification for why machine learning is appropriate and advantageous in addressing the research question.

>> We thank the reviewer for this suggestion and apologize for the lack of detail regarding our machine learning methods in the previous version. We have now significantly revised the Abstract, Results, and Discussion sections to provide a clear rationale and technical description of our approach. Specifically:

1. We have now specified algorithms in the abstract (Line 28-33): We trained four machine-learning models—Random Forest, Support Vector Machine, Logistic Regression, and Multilayer Perceptron—to discriminate between infected and uninfected plants based on microbiota composition, capturing both linear and non-linear relationships. The Random Forest classifier achieved maximal accuracy, while the Support Vector Machine demonstrated the most robust and consistent performance across different evaluation approaches.

2. We have significantly revised the 'Accurate microbiome-based prediction of plant infection status using machine learning models' section (Line 258-289) to provide a clearer and more detailed description of the machine learning framework, including model selection, parameter optimization, and evaluation strategies. Figure 4: The workflow diagram has been revised to more clearly illustrate the machine learning pipeline.

3. We also significantly improved the Method section 'Machine learning analysis' (Line 595-664) and added a comprehensive description of the training and validation strategies, including nested cross-validation for hyperparameter optimization and three complementary evaluation approaches (train–test split, 5-fold cross-validation, and leave-one-out cross-validation) to assess robustness and minimize overfitting. A summary of the optimized hyperparameters for all classifiers has been included in Supplementary Table 6.

4. From the previous version, we included evaluation of the machine learning methods by comparing them to other feature selection strategies such as Wilcoxon rank-sum test and LEfSe (Line 337-342). In this version, we also added a discussion of different strategies in predicting plant infection status from microbiome data (Line 518-529). This section justifies our approach by highlighting machine learning's ability to capture multivariate, non-linear

patterns and generalize new data. Additionally, we highlighted the limitations of our study and suggested future research directions.

We believe these revisions provide the necessary clarity and justification requested, and we thank the reviewer again for helping us improve the robustness of our manuscript.

2. Four machine learning methods are mentioned, but the rationale for selecting exactly four is unclear, and no discussion is provided regarding other approaches such as deep learning. The authors should explain the criteria for method selection and consider either justifying why deep learning was not applied or discussing its potential relevance.

>> We thank the reviewer for this suggestion. We have now revised the Result section (Line 261-268) to clarify our rationale of choosing these methods as indicated in the response to the above comment.

We also revised the Discussion section to add the potential relevance of deep learning strategies and the limitation of our dataset:

Line 527-532: However, given our relatively modest sample size (337 samples), we did not employ emerging deep learning architectures such as convolutional neural networks or transformers. These strategies generally require significantly larger datasets to achieve high predictive power[50], making classical classifiers a more appropriate and interpretable choice for this study. Future studies with larger sample sizes could benefit from exploring deep learning frameworks for microbiome-based disease prediction.

3. Figure 1a does not convey meaningful information, and the description of the Arabidopsis population dataset is insufficient. The figure should be revised to present interpretable and relevant data, such as sample distributions or phenotype summaries, and the dataset should be described in detail, including population size, genotypes, growth conditions, and preprocessing steps.

>> We thank the reviewer for this suggestion. We have now revised Figure 1a to include more details about our dataset. The revised figure now consists of three panels (a–c), showing the sampling locations, dataset distribution, and sample processing workflow.

4. The machine learning pipeline shown in Figure 4a is incomplete and does not reflect a full analytical workflow. The figure and accompanying text should be expanded to include all steps, from data preprocessing and feature selection to model training, evaluation, and interpretation, ensuring the pipeline is complete and reproducible.

>> We thank the reviewer for this insightful suggestion, which significantly improved the clarity of the machine learning part for broader readership. We have now re-designed Figure 4 to include all steps of the analytical workflow: including data preprocessing, hyperparameter optimization, feature selection via RFECV, model training and evaluation

across three validation schemes (70:30 split, 5-fold CV, and LOO CV), and model interpretation via feature importance scores. All methodological details are provided in the Methods section (lines 595-664), with a concise summary included in the Figure legend. All scripts are deposited in the GitLab repository as indicated in our response to the next point.

5. The code provided on the project website is unclear and appears to include only plotting scripts. The authors should provide complete, well-documented scripts covering data preprocessing, model training, evaluation, and visualization, along with a README file to guide reproduction of the results.

>> We thank the reviewer for this comment. We consider reproducibility to be of paramount importance, so we documented the GitLab repository (https://gitlab.nfdi4plants.de/zmbp/MLAraHDCom) with detailed and well-annotated scripts from all steps, and we included separated README files for every analysis. We realize that the original folder structure may have been confusing so that the reviewer may not have seen them. This has now been reorganized for clarity. Specifically:

1) **All scripts used for data analysis and figures plotting are now organized within the analysis_scripts/ directory in a clear and structured manner.**
2) **All datasets used for the analyses are provided in the data/ directory. External datasets (e.g., sequencing data) are clearly referenced and linked within the corresponding README files.**

We believe these changes, along with the updated README files, now provide a clear and intuitive guide for reproducing our results.

6. Genome analysis suggests potential terpene biosynthesis, but there is no direct evidence supporting antimicrobial activity. The authors should provide experimental or bioinformatic validation linking terpene pathways to antimicrobial compounds or explicitly present these results as preliminary and hypothesis-generating.

>> We thank the reviewer for this insightful comment. We fully agree that direct experimental evidence linking these specific terpene biosynthetic gene clusters to antimicrobial activity is lacking. We have now revised the Results and Discussion to present these genomic findings as preliminary and hypothesis-generating:

Line 437-441: While these genomic insights suggest that terpenoid compounds may be involved in the ecological fitness of *Cystofilobasidium*, their specific role in suppressing *Albugo* remains a preliminary hypothesis. These identified pathways provide a foundation for future functional studies to characterize the specific metabolites involved in this biocontrol activity.

Line 541-545: Although direct experimental evidence for their antimicrobial activity is currently lacking, these metabolites serve as strong candidates for mediating microbial competition and suppression. Future work involving metabolomics or gene knockout studies will be essential to confirm whether these specific terpenoids are the primary drivers of the observed plant protection.

We believe this more cautious interpretation accurately reflects the current scope of our data.

Reviewer #1 (Remarks on code availability):

The code provided on the website is not clear; it appears to include only some plotting scripts.
>> We apologize again for the unclear organization of our scripts. We hope our current structure of the GitLab repository (https://gitlab.nfdi4plants.de/zmbp/MLAraHDCom) is acceptable as described above.

Reviewer #2 (Remarks to the Author):

The additional bioinformatic analyses are convicing and confirm the value of the ML approach. Overall, the study is robust and can serve as a blueprint for upcoming research aimed at implementing ML for the exploration of microbiomes.

>> We are grateful for the reviewer's encouraging comments, and we also thank the reviewer for all insightful suggestions, which refined our machine learning pipeline and significantly improved the overall robustness of the study.

Minor suggestions:

Figure 4 combines an overview of the analysis method and table elements. It might be better to split this into a clean schematic overview and include detailed parameters shown in panel "b" as a supplementary table.

>> We thank the reviewer for this nice suggestion. We have now revised Figure 4a to include more details of the machine learning workflow, including data preprocessing, hyperparameter optimization, feature selection, model training, evaluation, and interpretation. The detailed performance metrics previously shown in panel (b and c) have been moved to a new (Supplementary Figure 4). This separation improves clarity and allows readers to follow the analytical workflow without distraction from detailed numerical results.

Panel "a" in Figure 6 could be improved to become visually more appealing. Particularly the image sizes showing the plants could be increased. Letters indicating statistical significance in panel "g" are much smaller than those in the other panels of the same figure.

>> We thank the reviewer for these suggestions. Figure 6a has been revised to improve visual clarity, including increasing the size of the plant images. The font size of the statistical significance letters in panel g has also been increased to ensure consistency across all panels.

Reviewer #2 (Remarks on code availability):

The code was properly deposited and the additional information provided on gitlab facilitates reproducibility.
>> We thank the reviewer for the positive evaluation of our code availability.

Reviewer #3 (Remarks to the Author):

The authors have carefully addressed the major and minor concerns raised during the first round of review. In particular, the revisions substantially clarify the rationale and implementation of the machine learning framework, improve transparency of model validation and feature selection, and better integrate the computational analyses with the experimental biocontrol assays. Overall, the revised manuscript is clearer, more balanced, and significantly strengthened compared to the previous version.
>> We sincerely thank the reviewer for the excellent suggestions, which significantly strengthened the conclusion and improved the clarity of this manuscript.

I have no remaining major concerns and support publication of the manuscript in its current form. I list below a few minor, optional points that the authors may consider addressing for further clarity, but none of these affect the main conclusions of the study.

Clarification of the added value of machine learning.

The revised text now more clearly positions the machine learning approach relative to conventional differential abundance and biomarker analyses. For maximal clarity to a broad readership, the authors may consider adding one or two concise summary sentences in the Discussion explicitly highlighting when and why the machine learning approach provides complementary or superior insights compared to traditional methods.

>> We thank the reviewer for this suggestion. We have revised the Discussion to explicitly highlight when and why machine learning outperforms conventional methods (Line 518-532). We clarify that machine learning captures coordinated community-level shifts by detecting patterns across many taxa simultaneously and provides predictive power on new samples — advantages not offered by conventional tools like LEfSe. We further contextualize our classifier choice relative to deep learning approaches, which require larger sample sizes than available in our study.

In summary, this is a well-executed and timely study that convincingly demonstrates how microbiome-informed machine learning can guide the identification of biocontrol agents in natural plant populations. I am pleased to recommend the manuscript for acceptance.

>> We thank the reviewer again for the positive evaluation of our study.

REVIEWERS' COMMENTS

Reviewer #1 (Remarks to the Author):

No

Reviewer #1 (Remarks on code availability):

No

Reviewer #2 (Remarks to the Author):

I have only raised mionor points in the previous version, and I'm happy with how they were addressed.

>> **We thank the reviewer for all valuable suggestions, which significantly improved our manuscript.**

Reviewer #2 (Remarks on code availability):

I didn't check this later on

Reviewer #3 (Remarks to the Author):

The authors have carefully addressed my previous concerns. The revised manuscript now provides a substantially clearer description of the machine-learning framework, including the algorithms used, model-selection rationale, validation strategies, hyperparameter optimization, and code availability. The revised figures and expanded Methods also improve the transparency and reproducibility of the computational analyses. I also appreciate the more cautious interpretation of the terpene-related genomic findings, which are now appropriately presented as preliminary and hypothesis-generating.

I have no remaining major concerns. I only suggest two minor points for clarity. First, the authors should explicitly state whether RFECV-based feature selection was performed strictly within the training folds during cross-validation, to avoid any potential information leakage.

>> We thank the reviewer for this helpful comment. We specified in the Approach 2 (Line 545-547) that the RFECV-based feature selection was performed strictly within the training set during cross-validation, so no information from the test data informed feature selection and data leakage was avoided. Our third approach (LOO-based) applied RFECV to the full dataset to maximize sample usage and the primary aim was feature discovery, with model-performance metrics treated as a secondary outcome. To clarify this, we have added the following sentence to the Methods (Approach 3):Line 557-560: Unlike the second approach, in which RFECV was applied strictly to the training set, this approach applied RFECV to the full dataset to maximize sample usage, with the primary aim of feature discovery and performance metrics serving as a secondary outcome.

Second, unless additional metabolite-level or functional evidence is provided, the wording around terpene-related antimicrobial activity should remain cautious; for example, “potential candidates” may be preferable to “strong candidates”.

>> We thank the reviewer for the suggestion and agree that more cautious wording is appropriate in the absence of metabolite-level evidence. In line with this suggestion, we have stated in the result section that the specific role of the predicted terpene pathways in suppressing *Albugo* "remains a preliminary hypothesis" and that "these identified pathways provide a foundation for future functional studies." We have also replaced 'strong candidates' with 'potential candidates' wherever applicable to ensure consistent cautious wording throughout."

Overall, the revision has substantially strengthened the manuscript, and I support publication after these minor clarifications.

>> We thank the reviewer again for the constructive and encouraging assessment of our manuscript.
